# Multiple linear regression modeling with values below a lower limit of quantification – a statistical method comparison

**DOI:** 10.1186/s12874-026-02770-y

**Published:** 2026-01-17

**Authors:** Lorena Hafermann, Isao Yokota, Linda Kalski, Bernd Wolfarth, Carolin Herrmann

**Affiliations:** 1 Institute of Biometry and Clinical Epidemiology, Charité - Universitätsmedizin Berlin, Charitéplatz 1, 10117 Berlin, Germany; 2 Department of Biostatistics, Graduate School of Medicine, Hokkaido University, N15, W7, Kita-ku, 060-8638 Sapporo, Japan; 3 Department of Sports Medicine, Charité - Universitätsmedizin Berlin, Charitéplatz 1, 10117 Berlin, Germany; 4 Institute of Sports Science, Department of Sports Medicine, Humboldt-Universität zu Berlin, Unter den Linden 6, 10117 Berlin, Germany; 5 Mathematical Institute, Faculty of Mathematics and Natural Sciences, Heinrich Heine University, Universitätsstraße 1, 40225 Düsseldorf, Germany

**Keywords:** Statistical method comparison, Left censoring, Lower limit of quantification, Regression model

## Abstract

**Background:**

Missing values occur in almost all real-world medical data. Sometimes, more information is available for the missing values due to technical measurement limits. This was also the case for some sports medical data set where several laboratory measurements below a lower limit of quantification (LLOQ) were faced and supposed to be used in a multiple linear regression model. When studying the literature, the problem arises in several disciplines (environmental epidemiology, pharmacokinetic studies etc.) and different statistical methods are suggested. However, only very limited work on a method comparison is available, especially in the multivariable linear regression settting.

**Methods:**

Therefore, we compare statistical methods for addressing values below a LLOQ in multiple linear regression modeling by a simulation study. We consider both the case that the variable below the LLOQ is among one of the independent variables and that it is the dependent variable in the regression model. We also vary different underlying assumptions, such as distributions, sample sizes, proportions of missing values, correlations, or linearity assumptions.

**Results:**

Overall, the two compartment model showed the best performance in terms of bias and coverage when the LLOQ occurred in the independent variable and no big collinearity issue was present. When the variable subject to the LLOQ is the dependent variable, tobit showed the lowest bias and highest coverage for censoring proportions up to 0.8.

**Conclusion:**

When facing a data set with values below a lower limit of quantification and a multiple linear regression model is chosen as analysis model, a conscious choice for dealing with those left-censored data should be made. In this article, we provide guidance on the performance of different established methods.

**Supplementary Information:**

The online version contains supplementary material available at 10.1186/s12874-026-02770-y.

## Background

Missing values are present in most data sets. In case missing values occur (completely) at random, there exist statistical methods to address them. However, there exist also values in data sets that are declared to be below some lower technical limit of quantification and are therefore missing. Those data are clearly not absent due to randomness, but related to their small values that cannot be detected with enough certainty. They occur in different types of data sets, e.g., clinical trials, observational studies but also related to different topics. Our work is motivated by the Ü45-check study [1, 2]. The Ü45-check study was supposed to determine the need for prevention and rehabilitation in Germany. More precisely, a preventive health examination conducted by physicians was compared to a questionnaire survey. In the related data set, one variable is the biomarker c-reactive protein (CRP), which is a left-censored variable due to a technical detection limit: values below a threshold of 0.6 mg/l could not be measured precisely enough such that all those were presented as “$<0.6$”. In the Ü45-check study, this applied to 26% of the participants. Nonetheless, the variable CRP was supposed to be used as independent variable in a multivariable regression model.

The terminology for the situation described above is very versatile: Examples are variable below a lower limit of quantification (BLOQ variable), left-censored variable, limited variable and nondetects [3, 4]. In the following, we use especially the terms BLOQ variable and left-censored variable interchangeably. Literature for dealing with values below a technical measurement limit in simple statistical testing settings, where we denote the threshold by lower limit of quantification (*LLOQ*), developed in parallel in different application areas, e.g., in clinical trials, genetic data and environmental data. Deleting those values happens from time to time but is almost always not a good practice. One strategy in pharmacokinetic (PK) studies is to substitute values below *LLOQ* with one half of the reporting limit [5]. Its limitations are already discussed in the literature with respect to underlying distributions etc. [4]. Keizer et al. [6] list and compare different methods addressing BLOQ variables in PK studies, among them also a likelihood-based approach. Johnson [7] gives also an overview on the treatment of BLOQ variables in PK studies. For environmental data, an overview with details on analysis and reporting BLOQ variables is given by Helsel [4]. He primarily recommends three approaches: First, he suggests non-parametric survival analysis procedures (i.e. Kaplan-Meier estimation) for data with censoring proportions of less than 50%. Applying survival analysis methods go back to Ware et al. [8] with the idea of transforming left- into right-censored data [9]. Second, for sample sizes of at least 50 and a censoring proportion of 50–80%, he recommends maximum likelihood estimation going back to [10, 11], e.g. censored regression methods. Those methods require the assumption that the selected distribution presents the observed data correctly. Third, he mentions regression on order statistics if the sample size is below 50 and a censoring proportion of 50–80% is prevalent. This includes binary methods, where values are classified into being above or below the *LLOQ*, and rank statistics in general. Due to the reduction to ranks, those methods can always be used but they come along with a loss in information. Note also that regression on ranks has a linearity problem. If the regression is linear in the predictor, then it is non-linear in the ranks and vice versa. Moreover, those methods lack interpretation of their regression coefficients. Method comparisons with the aim of estimating point estimates in the environmental data setting can be found in [12–18], and in [19–21] with a special focus on Bayesian statistics. Suzuki et al. [22] recently compared Bayesian with non-Bayesian estimation. Gleiss et al. [23] focused on the gene data setting with the additional challenge of no observable difference between true zeros and technical zeros.

Schisterman et al. 2006 [24] consider regression models, where the BLOQ variable enters as an independent variable. They compare different methods for addressing BLOQ values such as replacement by mean or zero. Having the BLOQ variable included as independent variable into a regression model is also considered in [25–28]. When considering the BLOQ variable as outcome variable and/or considering multivariable linear regression models with BLOQ variables, literature becomes much sparser or is even not existent. Jones et al. [29] consider left-censored multivariable regression modeling for environmental data using the methods of substitution of the BLOQ values with LLOQ and LLOQ/2. However, simulation studies comparing different situations in multivariable regression modeling like correlations, non-linearity and different distributions of the BLOQ variable are not yet available, neither for having a BLOQ variable as independent nor dependent variable. Similarly, software for addressing BLOQ variables is limited. The non-updated R-packages NADA (Nondetects And Data Analysis) and NADA2 are collections of methods described in [4]. Methods addressing the estimation of an area under the curve with present BLOQ values are implemented in the R-package BLOQ, which is based on Barnett et al. 2021 [30].

The purpose of our work is to conduct an in-depth method comparison for dealing with BLOQ variables in multivariable linear regression models – both as independent variable in a prediction model and dependent variable in a descriptive model. What is new next to the systematic comparison for dependent and independent BLOQ variables is our focus on different correlation structures, distributions of the BLOQ variable and potential non-linearity. The software implementation of the different methods is made freely accessible.

The manuscript is structured as follows: First, we describe the underlying multivariable linear regression models, including the motivating Ü45-check study, methods for addressing the BLOQ variables and strategies for performance evaluation. Even though the first case is inspired by the Ü45-check study, the results for both cases are general and can be applied to different applications. After introducing our simulation setup, the result section follows. We close with a discussion summarizing the most important findings as well as naming future potential extensions and a short conclusion.

## Methods

We start by describing the two regression models and methods addressing values below a lower limit of quantification, denoted by *LLOQ*. In order to assess the performance of the different methods handling variables with values below *LLOQ*, we conducted a simulation study. We considered two main data-generating models, which differ whether the BLOQ variable enters as independent or dependent variable. The models are intentionally simplified while still capturing the essential features of the underlying mechanisms.

### Model 1: BLOQ variable as independent variable

**Fig. 1:**
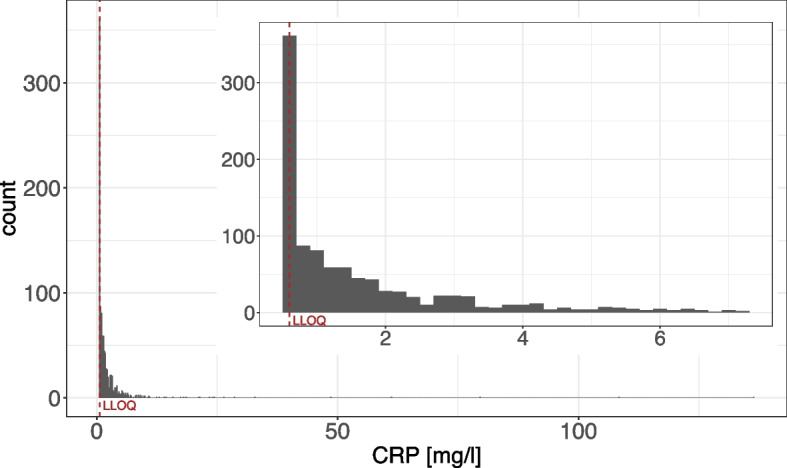
Histogram of c-reactive protein (CRP) values as in the Ü45-check study. The histogram in the upper right corner is focused on CPR values between 0 and 7.5 mg/l. LLOQ = lower limit of quantification

In Model 1, the BLOQ variable enters as independent variable. More precisely, it is the variable c-reactive protein (CRP), which can take on different distributions. This model is inspired by the Ü45-check study [1, 2], in which 26% of the values of the variable CRP were below *LLOQ* (cf. Fig. 1). It is known that CRP has an influence on cholesterol as, high CRP levels can indicate acute inflammatory reactions or tissue damage [31]. The aim of the model is to predict the influence of the variables CRP, sex and age on cholesterol.

The underlying true model for the sake of the simulation study is defined as

1 $$\begin{aligned} y_{cholesterol} = 40 + 3\cdot x_{age} - 1.5\cdot x_{sexmale} + 5\cdot x_{crp} + \epsilon , \end{aligned}$$

where $x_{age} \sim N(53, 4.17)$, $x_{sexmale} \sim Bin(1, 0.6)$, $x_{crp} \sim Beta(a,b)$ with differing values of *a* and *b* and $\epsilon \sim N(0,\sigma )$. The distributions of age and sex reflect realistic study values. The intercept as well as the regression coefficients were selected to produce cholesterol values that approximately match the empirical distribution in that data set. To evaluate the impact of skewness of the BLOQ variable, we used three different beta distributions for CRP: $x_{crp}\sim Beta(5,5)$ (centered), *Beta*(2, 8) (left-skewed) and *Beta*(8, 2) (right-skewed). The residual variance $\sigma$ was chosen to achieve adjusted $R^2$ values of 0.6 respectively 0.1, calculated a priori on a population data set of $n = 100,000$ for each setting. The exact values of $\sigma$ for all settings can be found in the Supplement S1.

### Model 2: BLOQ variable as dependent variable

In the Model 2, the variable affected by the technical detection limit is the outcome variable *y*. This model is not inspired by the Ü45-check study but is included for a comprehensive understanding of BLOQ variables in multivariable linear regression modeling. It can refer to situations such as estimating a treatment effect on a continuous outcome while adjusting for other variables. The underlying model is defined as

2 $$\begin{aligned} y = 2\cdot x_{treat} - 0.5\cdot x_1 + 3\cdot x_2 + \epsilon , \end{aligned}$$

where $x_{treat} \sim Bin(1,0.6)$, $x_1 \sim N(2,2)$, $x_2 \sim Beta(a,b)$ with differing values of *a* and *b* in each setting and $\epsilon \sim N(0,\sigma )$. Thereby $x_1$ and $x_2$ represent two additional independent variables, both describing fixed effects. In order to generate a left-, right-skewed or centered distribution of *y*, we used different distributions for $x_2$, i.e., $\beta (2,8), \beta (5,5),\beta (8,2)$. The values of $\sigma$ are determined in the same manner as described for the first model, simulated a priori on a population data set of $n = 100,000$ for each setting in order to archive the desired adjusted $R^2$ of 0.6 and 0.1 (cf. Supplement S1). Since the main focus of this model is the effect of the binary variable treatment, we did not consider a non-linear version of this model.

### Methods for addressing BLOQ values

In the following, the methods for handling BLOQ values, which are compared in our simulation study, are described with a focus on their applicability and integration within multivariable regression frameworks. In order to obtain a more comprehensive understanding of these methods’ performance in regression models, techniques previously criticized in the literature, such as deletion of BLOQ values or substitution, were likewise implemented [4, 6, 7, 30]. First, a general overview of the methods is provided, which is complemented with a formal, equation-based presentation.

- Discard: This method excludes all observations with BLOQ values from the analysis. While it is straightforward to implement, it reduces the sample size and can introduce bias as shown by Keizer et al.[6]. For this reason, several authors have advised against using this approach [4, 6, 30].
- Substitution: This approach substitutes BLOQ values with fixed values. Common choices are 0, the value *LLOQ* itself, *LLOQ*/2 (e.g., used in Frigerio et al. [32]), or $LLOQ/\sqrt{2}$. These substitutions are easy to implement but assign the same imputed value to all BLOQ observations, which may distort the distribution and underestimate variability. Substitution with zero will always underestimate the true values, if not all values are exactly zero. The substitution with LLOQ/2 implicitly assumes, that the BLOQ values are uniformly distributed and thereby follow a rectangular shape [5]. In most applications, a uniform distribution is rather unlikely. Therefore, one could argue that a triangular shape, referring to a straight line with a slope of one is a better fitting. The value, where half of the BLOQ values are left and right for a triangle is $LLOQ/\sqrt{2}$, which can be determined by solving the equation $\int _l^L cx\, dx = \frac{1}{2} \int _0^{LLOQ} cx\, dx$ for *l*, with a constant $c \in \mathbb {R}$ [5].
- Tobit regression (censored regression): Tobit regression models BLOQ values as left-censored observations using maximum likelihood estimation [4]. Accordingly, it is frequently referred to in the literature as ‘maximum likelihood estimation’. This method treats BLOQ values as an event occurring below a censoring threshold and is mathematically equivalent to fitting a censored regression model. It is only applicable when the BLOQ values occur in the dependent variable, and it assumes a known censoring limit like a known *LLOQ*. Mathematically, we define a continuous outcome variable $y_i^*$, which is linearly related to the independent variables $\textbf{x}_i$ via $y_i^* = \textbf{x}_i^\top \boldsymbol{\beta } + \varepsilon _i, \quad \varepsilon _i \sim \mathcal {N}(0, \sigma ^2),$ but it is only observed as
  $$\begin{aligned} y_i = \left\{ \begin{array}{ll} y_i^*, & \text {if } y_i^*> c, \\ c, & \text {if } y_i^* \le c, \end{array}\right. \end{aligned}$$
  where in our case the censoring limit *c* refers to the *LLOQ* and $i=1,...,n$. Under this model, the likelihood contribution for each observation *i* depends on censoring. For uncensored observations $(y_i^*> c)$, the likelihood is given by the normal density
  $$\begin{aligned} f(y_i \mid \textbf{x}_i) = \frac{1}{\sqrt{2\pi \sigma ^2}} \exp \left( -\frac{(y_i - \textbf{x}_i^\top \boldsymbol{\beta })^2}{2\sigma ^2} \right) . \end{aligned}$$
  For censored observations ($y_i^* \le c$), the likelihood contribution is the probability that $y_i^* \le c$, i.e., the cumulative distribution function of the normal distribution
  $$\begin{aligned} P(y_i \le c \mid \textbf{x}_i) = \Phi \left( \frac{c - \textbf{x}_i^\top \boldsymbol{\beta }}{\sigma } \right) . \end{aligned}$$
  Hence, the full log-likelihood function is given by
  $$\begin{aligned} \log L(\boldsymbol{\beta }, \sigma ) =& \sum \limits _{i \in \text {uncensored}} \left[ -\log \sigma + \log \phi \left( \frac{y_i - \textbf{x}_i^\top \boldsymbol{\beta }}{\sigma } \right) \right] \\&+ \sum \limits _{i \in \text {censored}} \log \Phi \left( \frac{c - \textbf{x}_i^\top \boldsymbol{\beta }}{\sigma } \right) , \end{aligned}$$
  where $\phi (\cdot )$ and $\Phi (\cdot )$ denote the standard normal probability density function and cumulative distribution function, respectively. To obtain the parameter estimates, this likelihood function is maximized using maximum likelihood estimation.
- K-nearest neighbors (kNN) algorithm: BLOQ values can also be imputed using a k-nearest neighbor (kNN) approach. For each BLOQ observation, the algorithm identifies the k most similar cases based on all observed independent variables and calculates the average of these neighbors’ corresponding values. In order to obtain values below *LLOQ*, the BLOQ value is then replaced by this value substituting *LLOQ*. This allows the imputed values to reflect variation in the surrounding data structure and incorporate multivariable information, making it more flexible than fixed substitution. For our simulation we used k = 5, representing a pragmatic trade-off between bias and variance.
- Kernel density imputation (KDE): Rather than assuming a specific distribution for the data, this method estimates the data distribution directly using kernel density estimation (KDE) based on the observed values. Specifically, it estimates the expected value of observations conditional on being below *LLOQ*. Barnett et al. (2021) [30] proposed a KDE-based approach for estimating the area under the curve (AUC) in longitudinal data subject to a technical detection limit. In our case the method can be applied to impute a single value for all observations below *LLOQ*. Formally, the density of the observed data $X_i$, is estimated as $\hat{f}(x) = \frac{1}{mh} \sum _{i = 1}^m K(\frac{x-X_i}{h}),$ where *m* is the number of values above *LLOQ*, $K(t) = \frac{1}{\sqrt{2}\pi } e^{-(1/2)t^2}$ is a Gaussian Kernel and $h = 1.06 \cdot \hat{\sigma } \cdot m^{1/5}$ is the bandwidth calculated according to Silverman’s rule of thumb [33]. The estimated density$\hat{f}(x)$ is then used to iteratively compute the expected value conditional on being below *LLOQ* represented by $k_{j} = E_{\hat{f}}(X|X <LLOQ)$, where *j* refers to the respective iteration step. The iterative process starts with the density estimated from the observed data above *LLOQ* and is repeated until convergence, i.e., $|k_j - k_{j-1}| <\epsilon$, with $\epsilon = 10^{-5}$ in our implementation. The $k_j$ from the final iteration is used to impute the BLOQ values. In our single time-point scenario, this results in a single imputed value applied to all BLOQ observations. The $k_j$ can be estimated by $\sum _{\ell \in \mathcal {S}}(x_\ell \cdot y_\ell )/\sum _{\ell \in \mathcal {S} } x_{\ell }$, where $\mathcal {S}$ equals the set of all *m* values above *LLOQ* and all $k_j$ from all iterations before.
- Two compartment model: This approach is applicable when BLOQ values occur in independent variables. The BLOQ variable is split into two components: a binary indicator variable representing whether the observation is below the technical detection limit or not, and a continuous variable representing the observed value when it is above *LLOQ*. Both components are included in the regression model, allowing the influence of presence/absence and magnitude to be modeled separately. For a simple univariable model with an outcome *y* and an independent variable *x*, which is subject to BLOQ values, this means that *x* is split into a binary variable
  $$\begin{aligned} x_{bin} = \left\{ \begin{array}{ll} 0, & \text {if } x_i> LLOQ, \\ 1, & \text {if } x_i \le LLOQ, \end{array}\right. \end{aligned}$$
  $i=1,...,n$, and continuous variable $x_{cont}$, where all BLOQ values are set to any value between 0 and *LLOQ*. This results in the model $y = x_{bin} + x_{cont} + \epsilon ,\ \epsilon \sim N(0,\sigma )$.

Beyond the methods outlined above, additional approaches can be found in the literature, though they are not readily applicable to our setting of multivariable regression modeling. Another straight forward method is to dichotomize the BLOQ variable. However, this addresses a different research question and requires a medically relevant boundary. In any case, such dichotomization includes a loss of information. Moreover, more sophisticated methods based on Bayesian statistics are available, such as the approach proposed by Suzuki et al. (2020) [22], which is designed to estimate summary statistics, including the mean and standard deviation. Another frequently used method is regression on order statistics, which models censored data by fitting a parametric distribution to the observed values and then predicting the censored observations based on their expected order in that distribution [4]. This approach can as well be used for deriving summary measures but cannot be directly extended to multivariable regression modeling, as it does not incorporate covariate effects into the estimation process. Similarly, the Akritas–Theil–Sen (ATS) estimator is a robust, nonparametric method that generalizes the Theil–Sen slope estimator to handle censored data [4]. While ATS is resistant to outliers and does not require strong distributional assumptions, it can only be applied to univariable regression models and is therefore not suitable for modeling relationships involving multiple covariates. A further interesting approach is the one recently suggested by Hawkins and Esquivel [34]. The underlying idea is to use a quantile-quantile plot based on the observed values and by application of a QQ regression to retrieve estimates for the mean and standard deviation while considering the “effective” sample sizes. On the one hand, it can be seen as a simple alternative to the KDE approach. On the other hand, it depends strongly on whether the values follow or can be transformed to a normal distribution which has been censored as well as on the underlying sample size. Moreover, we did not include models such as Tweedie regression, e.g. [35], as we have an interval of values missing and no peak at zero, Hurdle models [36] or zero inflated models as we have no count data.

### Simulation Settings

In total, we considered five potential settings, varying in the skewness of the BLOQ variable, the correlation and linearity, which are listed in Table 1. Initially, all variables were simulated independently of each other without any correlation. In a second step, correlations were added between $x_{crp}$ and $x_{age}$ of 0.8 in Model 1 and between $x_{treat}$ and $x_1$ of 0.6 in Model 2 to test the effect of moderate to strong correlations. In order to assess the methods in a setting where the linearity assumption is not met, we additionally considered adding a quadratic term of CRP in Model 1:

3 $$\begin{aligned} y_{cholesterol} =\:& 40 + 3\cdot x_{age} - 1.5\cdot x_{sexmale} \\&+ 3\cdot x_{crp} + x_{crp}^2 + \epsilon . \end{aligned}$$

The coefficients were chosen to match the distribution of cholesterol in the linear model.

**Table 1:** Overview of all five settings considered in our simulation study. Model 1 refers to the setting where the BLOQ variable enters as independent variable into the regression model and Model 2 to the case of the BLOQ variable entering as dependent variable into the model

Setting	Distribution	Correlation	Linearity	Models
S1	right skewed	no	linear	Model 1 and 2
S2	left skewed	no	linear	Model 1 and 2
S3	centered	no	linear	Model 1 and 2
S4	right skewed	yes	linear	Model 1 and 2
S5	right skewed	no	non-linear	Model 1

Simulation settings S1–S5 were applied to Model 1 and settings S1–S4 were considered for Model 2. For all settings, the coefficient of determination $R^2_{adj} \in \{0.1,0.6\}$, sample sizes of $n \in \{30,45,60,100,200,300,400,500,1000\}$ were simulated and the proportion of values below the lower limit of quantification was varied between $\{0.01,0.05,0.10,0.15,0.20,...,0.90,0.95\}$. For each model, sample size and proportion of values below the LLOQ, 1,000 simulation runs were performed. All simulations were conducted in R (Version 4.5.0). For the kNN approach, we used the VIM package in our computations [37].

### Performance evaluation

To the performance of the methods in the simulation study, we estimated bias, coverage, and mean squared error (MSE) for Model 1, and bias and coverage for Model 2. For Model 1, which aims at correctly predicting cholesterol levels, in a setting that includes an independent BLOQ variable, i.e. CRP, we were interested in the bias of CRP

$$\begin{aligned} \hat{\beta }_{CRP}-\beta _{CRP}. \end{aligned}$$

We have $\beta _{CRP} = 5$ in Model 1 (excluding S5). For the non-linear setting (S5), the true parameter $\beta _{CRP}$ was defined via the beta parameter of the linear model in the population data set with $n = 100,000$. In addition, the coverage was calculated, defined as the proportion of simulation runs in which the true value $\beta _{CRP}$ was included within the corresponding 95%-confidence interval. As predictive performance was also of interest for this model, we additionally calculated the mean squared error (MSE) defined on the population data sets. The MSE is given by

$$\begin{aligned} MSE = \frac{1}{n}\ \sum \limits _{i=1}^{n} (\hat{y}_i - y_i)^2, \end{aligned}$$

with sample size *n*.

For Model 2, which represents a descriptive model, we were interested in correctly estimating the effect of the treatment $\beta _{treat}$ on a dependent variable. Here, the dependent variable is the BLOQ variable. Similarly, the bias is defined as $\hat{\beta }_{treat}-\beta _{treat}$ and the coverage as the proportion of simulation runs in which the true value $\beta _{treat}$ was included within the corresponding 95%-confidence interval. As predictive performance was not of interest for this model, the MSE was not calculated.

## Results

**Fig. 2:**
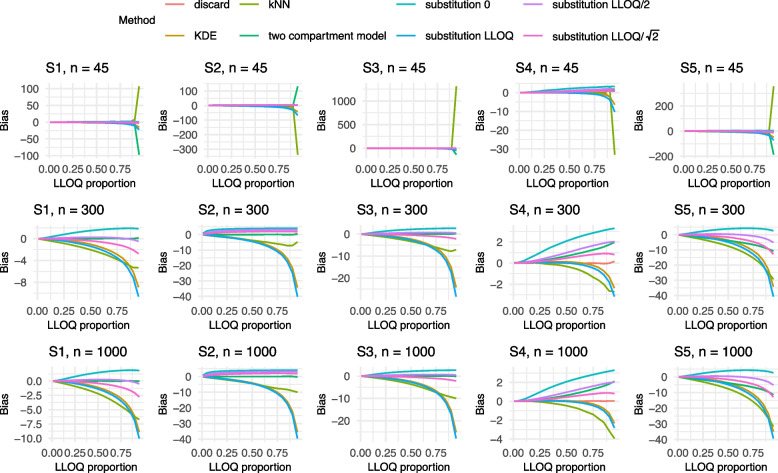
Results of the bias for Model 1 with an $R^2 = 0.6$ for Settings S1-S5 and $n= 45, 300, 1000$. The colors refer to the different methods addressing BLOQ values. LLOQ = lower limit of quantification

**Fig. 3:**
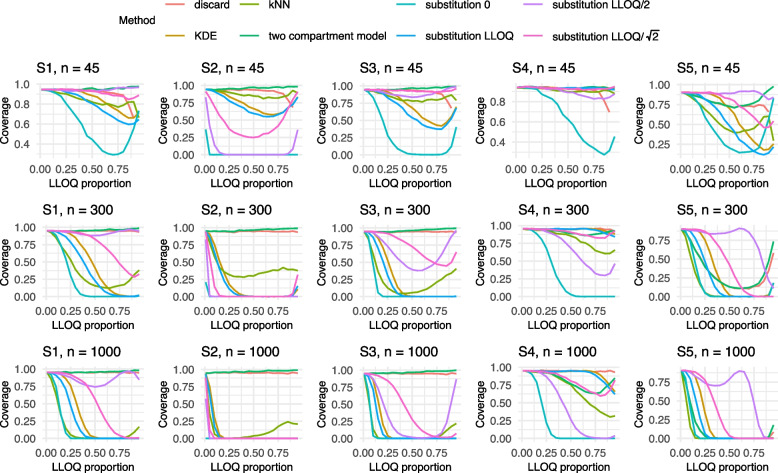
Results of the coverage for Model 1 with an $R^2 = 0.6$ for Settings S1-S5 and $n= 45, 300, 1000$. The colors refer to the different methods addressing BLOQ values. LLOQ = lower limit of quantification

**Fig. 4:**
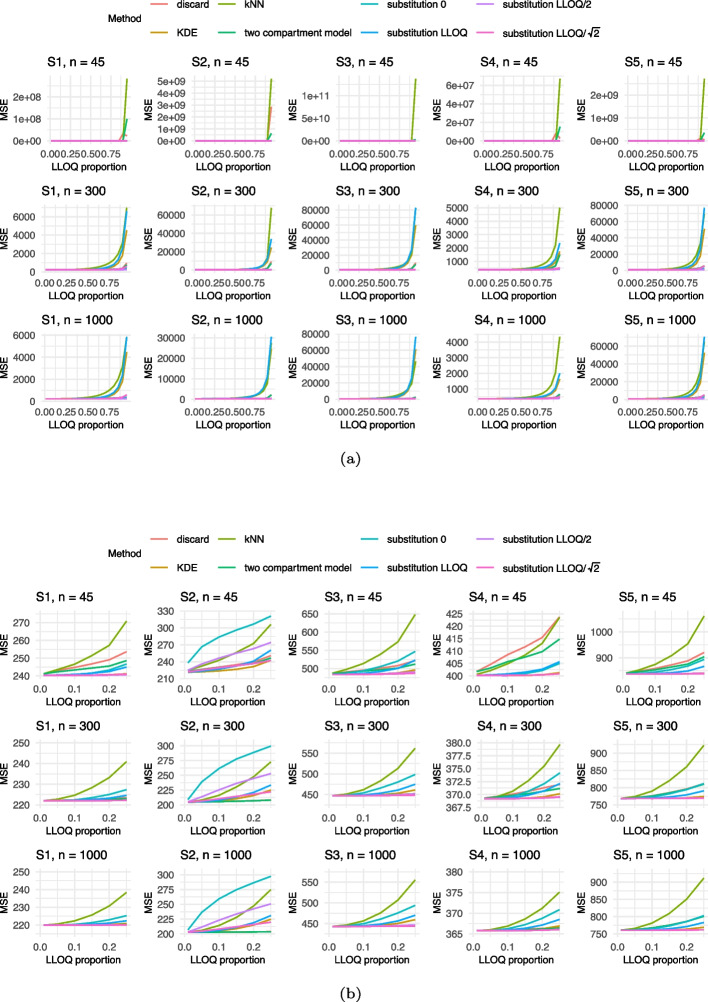
Results of the Mean Squared Error (MSE) for Model 1 with an $R^2 = 0.6$ for Settings S1-S5 and $n= 45, 300, 1000$ for (**a**) all LLOQ proportions and (**b**) restricted to LLOQ proportions from 0 to 0.25. The colors refer to the different methods addressing BLOQ values. LLOQ = lower limit of quantification

**Fig. 5:**
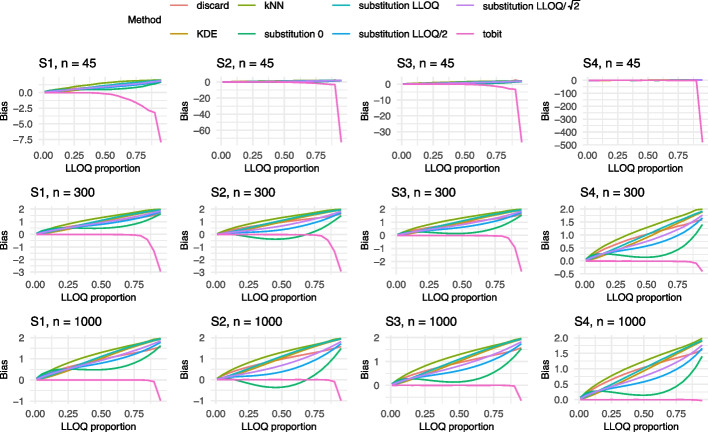
Results of the bias for Model 2 with an $R^2 = 0.6$ for Settings S1-S4 and $n= 45, 300, 1000$. The colors refer to the different methods addressing BLOQ values. LLOQ = lower limit of quantification

**Fig. 6:**
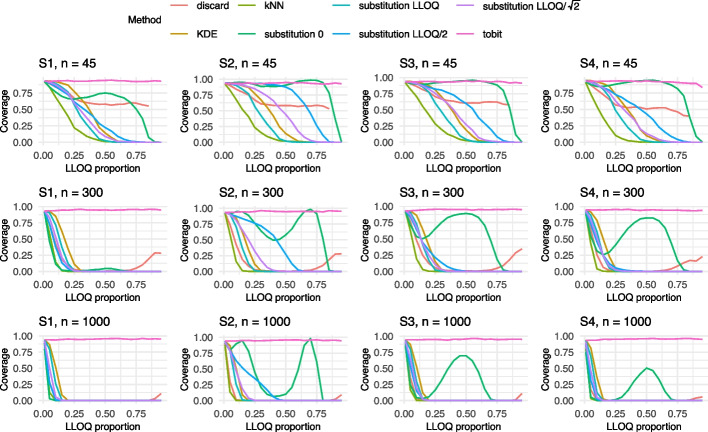
Results of the coverage for Model 2 with an $R^2 = 0.6$ for Settings S1-S4 and $n= 45, 300, 1000$. The colors refer to the different methods addressing BLOQ values. LLOQ = lower limit of quantification

Given the large number of scenarios, only the results for both models corresponding to an adjusted $R_{adj}^2$ of 0.6 and sample sizes $n \in \{45,300,1000\}$ are visualized in Figs. 2, 3, 4, 5 and 6 in this manuscript. All other results can found in the Supplement S2.

### Results of Model 1

Most methods exhibited an increase in bias as the proportion of values below the LLOQ increased (cf. Fig. 2). For a small sample size of n=45 and LLOQ proportions above 0.5, the bias became particularly high, due to the limited amount of data above the LLOQ. In contrast, the results for n=300 and n=1000 were more stable and showed similar patterns. Including a correlation between two of the independent variables (S3), less negative, but more positive bias, could be observed. As expected, the estimate under the discard method remained unbiased under Settings S1–S4 but displayed a bias comparable to the two compartment model under Setting S5. Notably, Setting S5, which differed from S1 by the inclusion of a nonlinear term, produced results that were largely consistent with S1. Across all settings and sample sizes, both the KDE and substitution with *LLOQ* exhibited a relatively high bias. Additionally, kNN showed considerable bias in Setting S4. In contrast, the two compartment model demonstrated the best performance in terms of bias for Settings S1–S3. For the models corresponding to an $R_{adj}^2$ of 0.1, very similar results were found with a slightly larger bias upwards (cf. Supplement S2.1.2).

With respect to coverage, the two compartment model demonstrated strong performance under Settings S1–S3, moderate performance in S4, and poor performance in S5 (cf. Fig. 3). Setting S5, characterized by a nonlinear effect, was particularly problematic, with most methods performing poorly; notably, substitution with *LLOQ*/2 yielded the best coverage in this setting. The simple substitution with *LLOQ* performed poorly in S1–S3, but its performance improved substantially in the presence of added correlation. The discard method exhibited generally good coverage across S1–S4, largely due to the widening of confidence intervals as a result of reduction of sample size. However, under the non-linear Scenario S5, its performance deteriorated markedly. Substitution with *LLOQ*/2 showed reasonable coverage in right-skewed distributions, but performed poorly in left-skewed scenarios, and failed to provide reliable coverage for centered distributions. It is noticeable that some of the curves are U-shaped, e.g., in the substitution with *LLOQ*/2 in the right-skewed data in S1, which is best visible for $n = 1000$. For low LLOQ proportions, the approximation by *LLOQ*/2 is still suitable but as the LLOQ proportion increases, the underlying assumption of a uniform distribution below LLOQ, which is implied by the substitution of *LLOQ*/2, is no longer valid. On the other hand, with very high LLOQ proportions, the confidence intervals become wider, which increases the coverage. Regarding the results for settings with an adjusted $R_{adj}^2$ of 0.1, it seems that decreasing the coefficient of determination yields a similar effect as decreasing the sample size (cf. Supplement S2.2.2). Notably, the outcomes for $R_{adj}^2$ of 0.6 with a sample size of n = 30 closely resemble those observed for $R_{adj}^2$ of 0.1 with n = 300.

In terms of MSE, the substitution method using *LLOQ*, as well as the kNN and KDE approaches, consistently exhibited the poorest performance across all scenarios. This trend was most pronounced for larger sample sizes (cf. Fig. 4). However, it is important to note that substantial differences in MSE between methods only arise when the LLOQ proportion exceeds 0.5. In most practical applications, lower LLOQ proportions are more relevant when considering the application of a multivariable linear model. Focusing on this more applicable range, substitution with zero yields the highest MSE, followed by kNN as can be seen in Fig. 4b. In contrast, KDE achieves relatively low MSE values at low LLOQ proportions. Unlike the findings for bias and coverage, the MSE results show minimal variation across different settings. One exception is observed under $R_{adj}^2$ of 0.1, where the MSE of the discard method increases noticeably.

### Results of Model 2

In contrast to the results from Model 1, the bias patterns under Model 2 differ substantially (cf. Fig. 5). Tobit regression displays a remarkably low bias across a wide range of LLOQ proportions. However, its performance deteriorates notably at higher LLOQ levels, likely due to the decreasing number of uncensored observations. Nevertheless, its overall behavior remains stable compared to other methods. Across most scenarios, a consistent positive bias is observed for nearly all methods, with the exception of substitution with zero, which tends to underestimate the bias. Interestingly, the bias profiles exhibit similar shapes across all simulation settings, suggesting a certain robustness of these patterns to underlying model specifications. Among all methods, kNN consistently shows the highest levels of bias. Given these findings, the tobit model stands out as the most reliable approach for bias reduction and can be recommended over alternative methods in the presence of censored data. Overall, the differences from the $R_{adj}^2 = 0.1$ setting are minor, with the main distinction being slight variations in the shape of the bias curves. When considering results under a reduced $R_{adj}^2 = 0.1$, tobit once again performs comparatively well, maintaining low bias across most scenarios. The substitution with zero exhibits a wavy bias pattern, while the discard method performs poorly, falling below the upper bias bounds observed for kNN. As in earlier observations, kNN and discard show a similar behavior. Overall, the differences from the setting with $R_{adj}^2 = 0.1$ are minor, showing similar bias curves (cf. Supplement S2.2.2).

With regards to the coverage, distinct U- and M-shaped patterns emerged across the scenarios (cf. Fig. 6). Notably, Settings S3 and S4 produced remarkably similar coverage outcomes. For small sample sizes, coverage results across settings S1 to S4 were largely consistent, suggesting limited sensitivity to the specific data-generating structure. Among the methods evaluated, kNN once again performed relatively well in terms of maintaining coverage. In contrast, discarding the BLOQ values showed a decline in performance at larger sample sizes, likely due to narrower confidence intervals that fail to account for censoring uncertainty. Overall, tobit regression demonstrated the most reliable coverage behavior across scenarios. Other methods performed poorly or only achieved acceptable coverage intermittently, limiting their applicability. When the adjusted $R_{adj}^2$ was reduced to 0.1, a general deterioration in coverage performance was observed. The improvement in coverage seen under $R_{adj}^2 = 0.6$ compared to $R_{adj}^2 = 0.1$ appeared to have a similar effect as a larger sample size. This suggests that increased noise in the data substantially hinders coverage accuracy, particularly for smaller samples. Nevertheless, tobit continued to perform comparatively well, whereas the remaining methods offered little consistency or reliability.

## Discussion

The goal of this work was a detailed comparison of different statistical methods for addressing values below a lower limit of quantification in multivariable linear regression modeling with a special focus on different underlying distributions, correlations and potential non-linearity. Initially, the motivation came from the Ü45-check study [2] where such a model with a BLOQ variable (here: CRP) as one of the independent variables in a multivariable linear regression model was planned to be used. As our literature review resulted in very sparse knowledge on the comparison of different methods for multivariable linear regression modeling, we decided to conduct the corresponding research. Hence, we both considered the case of the BLOQ variable entering as independent variable in a prediction model and the BLOQ variable entering as dependent variable in a descriptive model to provide an even broader overview.

In our simulation study, we varied different underlying assumptions such as data distributions, correlation, linearity and goodness of fit of the regression models as well as sample size and proportion of the LLOQ values. For addressing the BLOQ variable, we considered different methods. Note that some are only valid when the BLOQ variable enters as dependent variable (e.g., tobit model) or only as independent variable (e.g., the two compartment model). In the regression model where the BLOQ variable enters as independent variable, the two compartment model performed reasonably well with respect to our performance measures bias and coverage. The kernel density estimation had often a rather large bias. Despite the fact that discarding the BLOQ values is known to be unbiased in linear models if the BLOQ variable enters as the independent variable [38], this no longer holds true in the non-linear setting as well as when the BLOQ variable is the dependent variable. In addition, discarding the BLOQ values always leads to a loss of sample size and thus to a widening of the confidence intervals. Therefore, we generally argue against its use. Discarding has also performed particularly poorly in terms of the mean squared error. In Scenario 4, however, the discard method showed only small bias, whereas the two compartment approach exhibited larger bias. This can be explained by the strong correlation between CRP and age, which induced multicollinearity and distorted the role of the indicator variable. This situation represents a setting in which the BLOQ variable is correlated with other independent variables, for example in the presence of confounding. In such settings, the two compartment approach becomes inappropriate because the effect of the indicator is masked by collinearity and the adjustment is partly absorbed by other covariates, leading to biased estimates. Thus, discarding the BLOQ observations, although inefficient, may provide the least biased option in the presence of such correlation structures. This result is also consistent with critiques of the missing-indicator method [39], of which the two compartment approach can be regarded as a special case and which is known to yield biased estimates.

In the regression model where the BLOQ variable enters as dependent variable, the tobit model performed overall best with respect to the two performance measures bias and coverage. At larger sample sizes, no method except tobit showed acceptable coverage for LLOQ proportions greater than 0.25. Across all scenarios, kNN showed a low bias and coverage compared to the other methods. Therefore, we cannot recommend it.

Note that in general one has to keep in mind that not every proportion of LLOQ values is worth to address with a multivariable linear regression model. If the proportion of LLOQ values is very large, then we likely face other statistical problems which can be more severe. It can make sense to define an upper limit of the LLOQ proportion and otherwise consider different models, e.g., generalized additive models with splines. However, for the sake of completeness, we presented a wide range of proportions. Note that likewise, also the absolute LLOQ values could be reported on the x-axes. Moreover, we want to highlight that our multivariable regression models contain only a relative small number of independent variables. In application, often a larger number of variables is considered. Furthermore, in this work, we focused on a systematic comparison of a right-skewed, left-skewed and central distribution. Depending on future application scenarios at hand, a careful and realistic choice of the underlying distribution should be made. Note that we did not compare our results with non-parametric regression modeling techniques to remain fair in comparison measurements.

There exist multiple possibilities of extension for the described comparison. First, one could compare values below a lower and values above an upper limit of quantification. Second, there can also be situations where several variables face the problem of being left-censored, i.e., also both the dependent and an independent variable. Third, reporting limits might change over time (e.g., new measurement techniques), which in turn leads to different LLOQ values.

## Conclusion

When facing a data set with values below a lower limit of quantification and a multiple linear regression model is chosen as analyzing model, a conscious choice for dealing with left-censored data needs to be made. In this article, we provide guidance on the performance of different methods, which have not been compared in such a systematic manner before. In the case, where left-censoring occurs in an independent variable, the two compartment model seems to be a good starting point. When left-censoring occurs in the dependent variable, a tobit model seems to be a good starting point.

## Supplementary Information

Additional file 1. Additional information on the standard deviation of $\epsilon$ of the random error of the models and additional figures of the results can be found in the supplement.

## Data Availability

All data generating and analyzing codes are included in the supplementary information files of this published article.
