# Supplementary material for: Multiple linear regression modeling with values below a lower limit of quantification – a statistical method comparison
Source: BMC Med Res Methodol. 2026 Jan 17;26:18. doi: 10.1186/s12874-026-02770-y (PMC12853717; doi:10.1186/s12874-026-02770-y)
Supplement: Supplementary file 1 — Additional file 1. Additional information on the standard deviation of $\epsilon$ of the random error of the models and additional figures of the results can be found in the supplement. [file 12874_2026_2770_MOESM1_ESM.zip › Supplement_LLOQ_BMC.pdf]

Supplement
Multiple linear regression modeling with values below a lower limit of quantification – a statistical method comparison

Lorena Hafermann, Isao Yokota, Linda Kalski,
Bernd Wolfarth, Carolin Herrmann

S1 Additional Information

Table S1: For Model 1 and Model 2 the random error ϵ is normally distributed with $\epsilon \sim N(0, \sigma)$. We list the specific values of σ for each setting and model. Setting S5 was not used for Model 2.

	Model 1		Model 2	
Setting	$R^2 = 0.1$	$R^2 = 0.6$	$R^2 = 0.1$	$R^2 = 0.6$
S1	55.55	14.81	4.43	1.17
S2	52.48	14.22	4.36	1.18
S3	78.47	21.02	4.65	1.26
S4	71.19	19.10	2.88	0.77
S5	103.05	26.42	–	–

S2 Additional Figures

S2.1 Bias, coverage and MSE performance of Model 1: BLOQ variable as independent variable

S2.1.1 Results for $R^2_{adj} = 0.6$

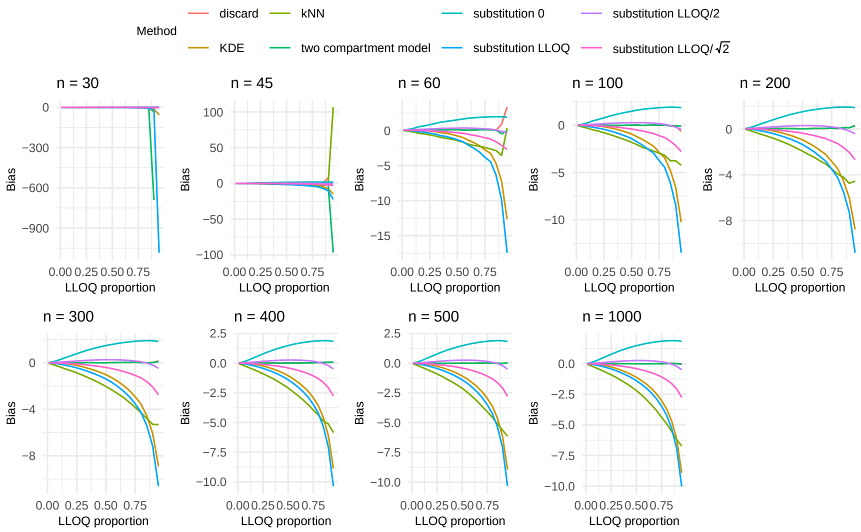

Figure S1: Results of the bias for Model 1 with an $R^2 = 0.6$ for Setting S1. The colors refer to the different methods addressing BLOQ values. LLOQ = lower limit of quantification.

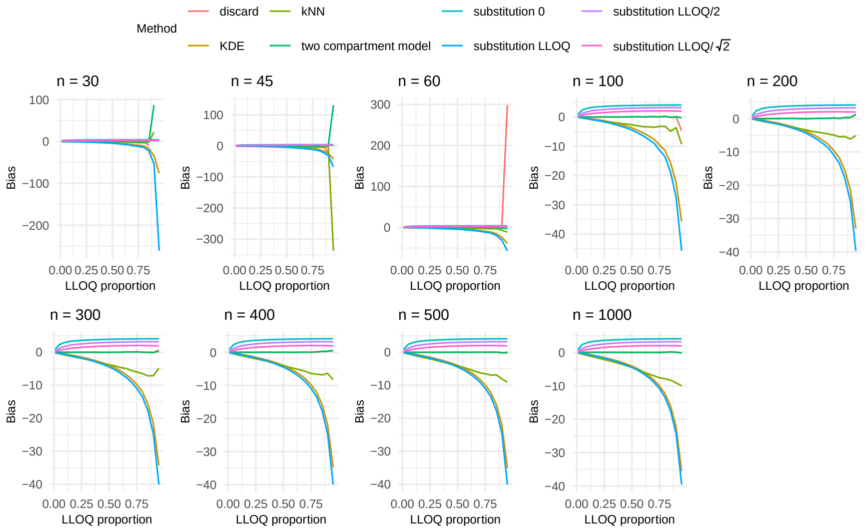

Figure S2: Results of the bias for Model 1 with an $R^2 = 0.6$ for Setting S2. The colors refer to the different methods addressing BLOQ values. LLOQ = lower limit of quantification.

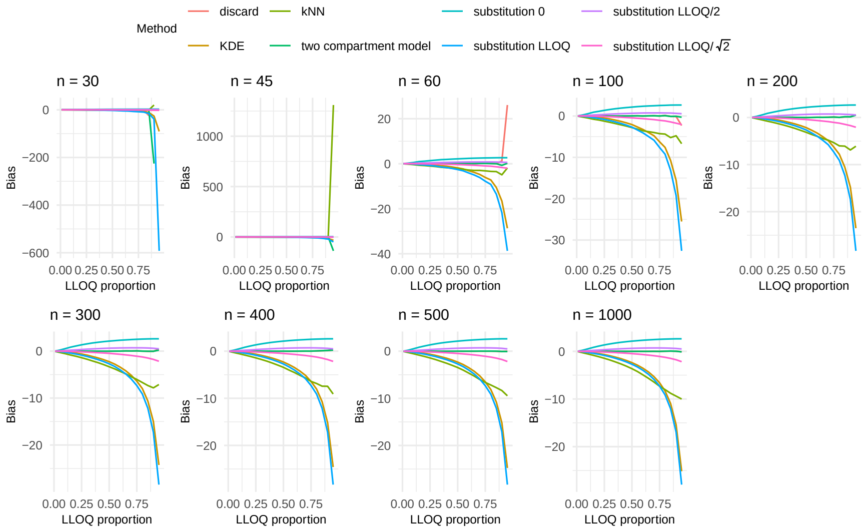

Figure S3: Results of the bias for Model 1 with an $R^2 = 0.6$ for Setting S3. The colors refer to the different methods addressing BLOQ values. LLOQ = lower limit of quantification.

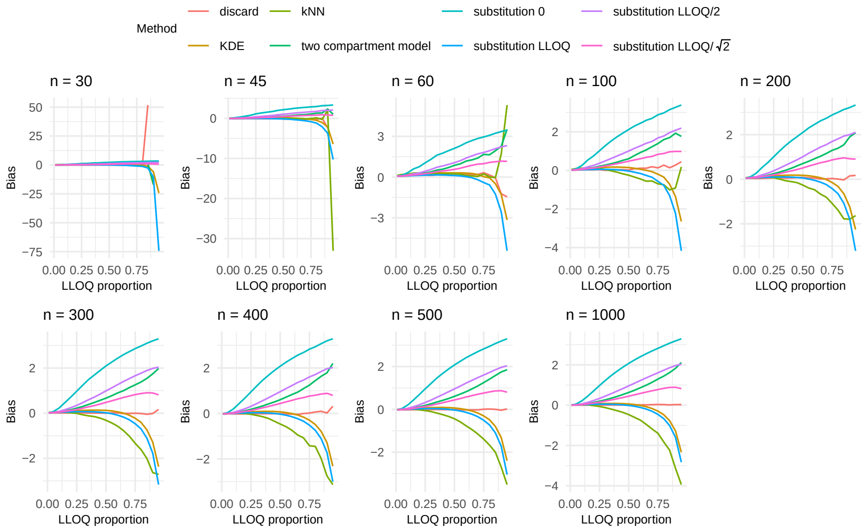

Figure S4: Results of the bias for Model 1 with an $R^2 = 0.6$ for Setting S4. The colors refer to the different methods addressing BLOQ values. LLOQ = lower limit of quantification.

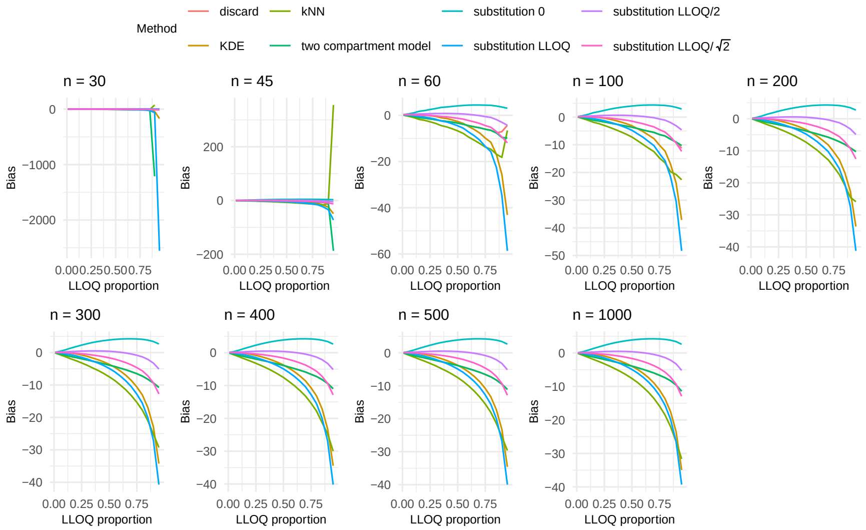

Figure S5: Results of the bias for Model 1 with an $R^2 = 0.6$ for Setting S5. The colors refer to the different methods addressing BLOQ values. LLOQ = lower limit of quantification.

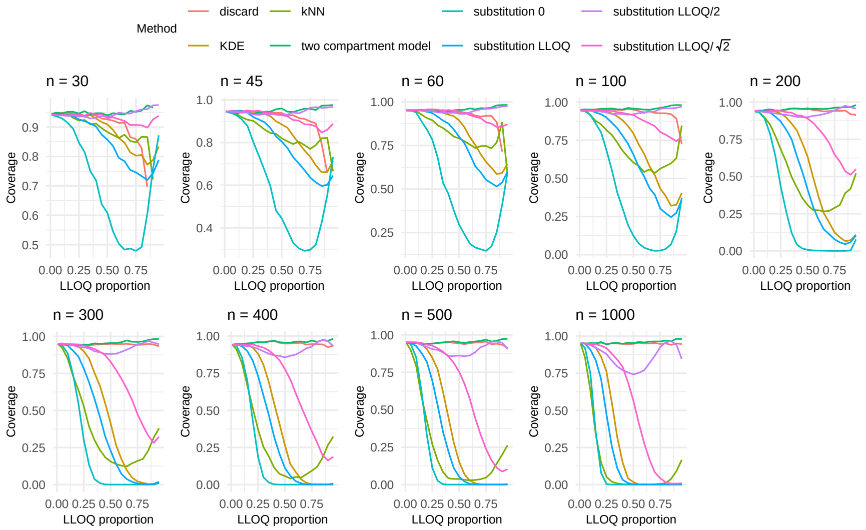

Figure S6: Results of the coverage for Model 1 with an $R^2 = 0.6$ for Setting S1. The colors refer to the different methods addressing BLOQ values. LLOQ = lower limit of quantification.

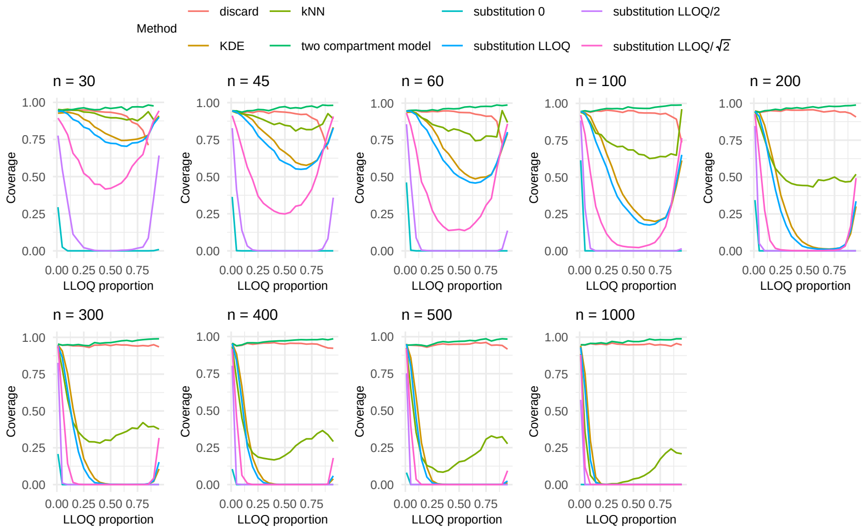

Figure S7: Results of the coverage for Model 1 with an $R^2 = 0.6$ for Setting S2. The colors refer to the different methods addressing BLOQ values. LLOQ = lower limit of quantification.

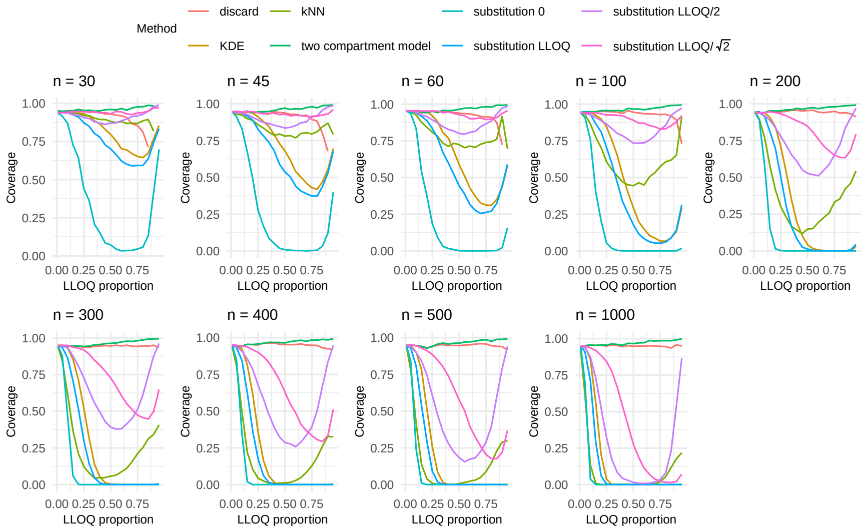

Figure S8: Results of the coverage for Model 1 with an $R^2 = 0.6$ for Setting S3. The colors refer to the different methods addressing BLOQ values. LLOQ = lower limit of quantification.

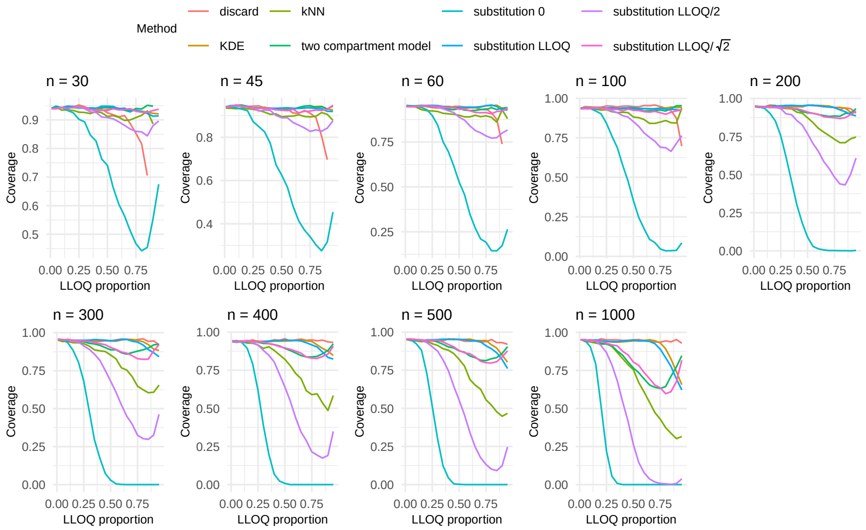

Figure S9: Results of the coverage for Model 1 with an $R^2 = 0.6$ for Setting S4. The colors refer to the different methods addressing BLOQ values. LLOQ = lower limit of quantification.

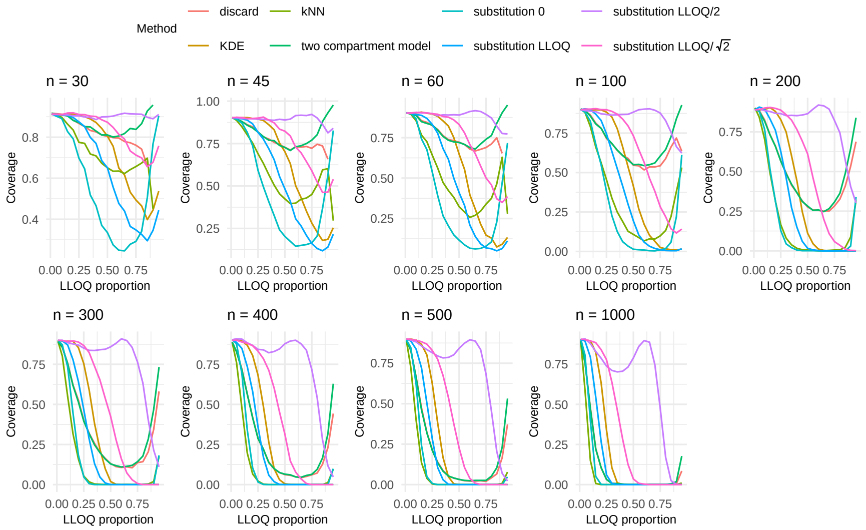

Figure S10: Results of the coverage for Model 1 with an $R^2 = 0.6$ for Setting S5. The colors refer to the different methods addressing BLOQ values. LLOQ = lower limit of quantification.

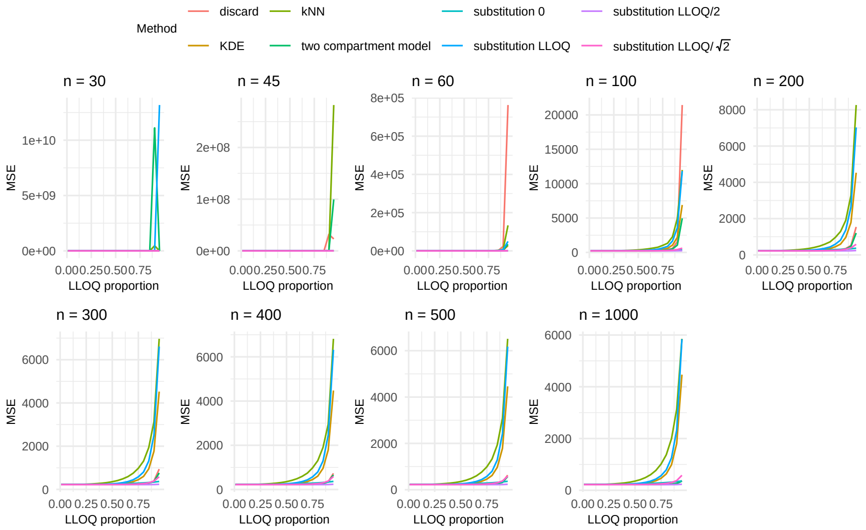

Figure S11: Results of the MSE for Model 1 with an $R^2 = 0.6$ for Setting S1. The colors refer to the different methods addressing BLOQ values. LLOQ = lower limit of quantification.

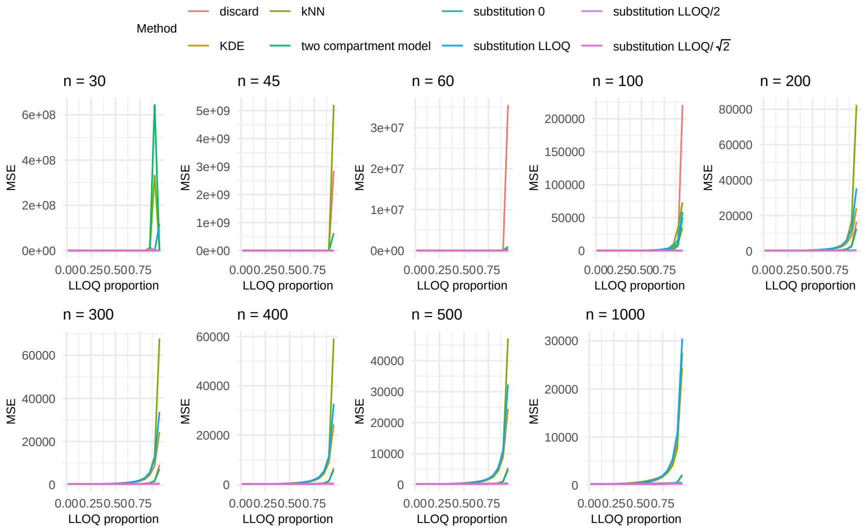

Figure S12: Results of the MSE for Model 1 with an $R^2 = 0.6$ for Setting S2. The colors refer to the different methods addressing BLOQ values. LLOQ = lower limit of quantification.

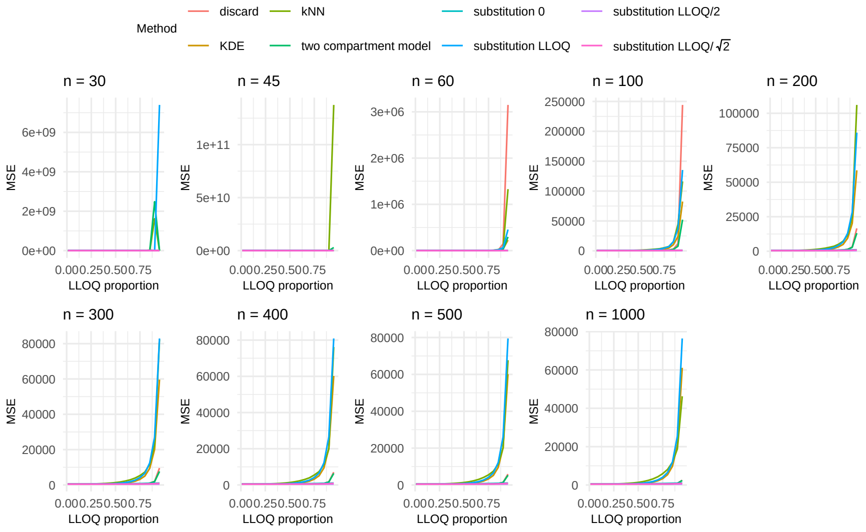

Figure S13: Results of the MSE for Model 1 with an $R^2 = 0.6$ for Setting S3. The colors refer to the different methods addressing BLOQ values. LLOQ = lower limit of quantification.

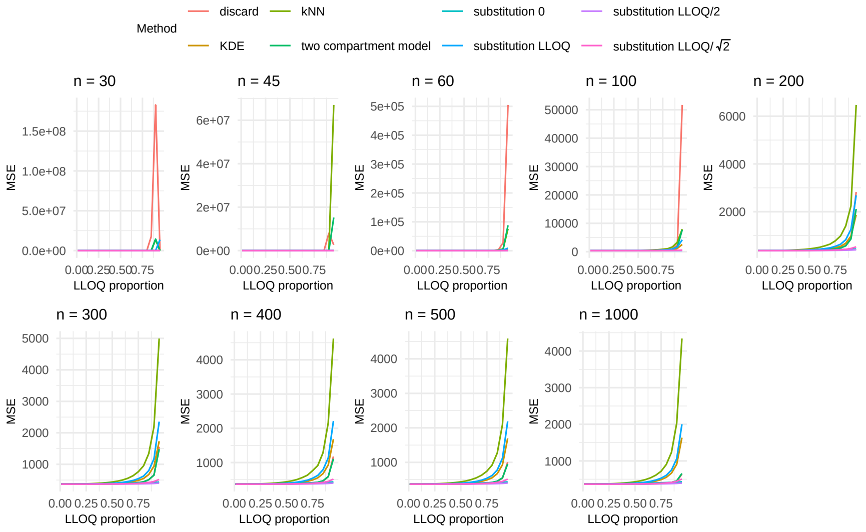

Figure S14: Results of the MSE for Model 1 with an $R^2 = 0.6$ for Setting S4. The colors refer to the different methods addressing BLOQ values. LLOQ = lower limit of quantification.

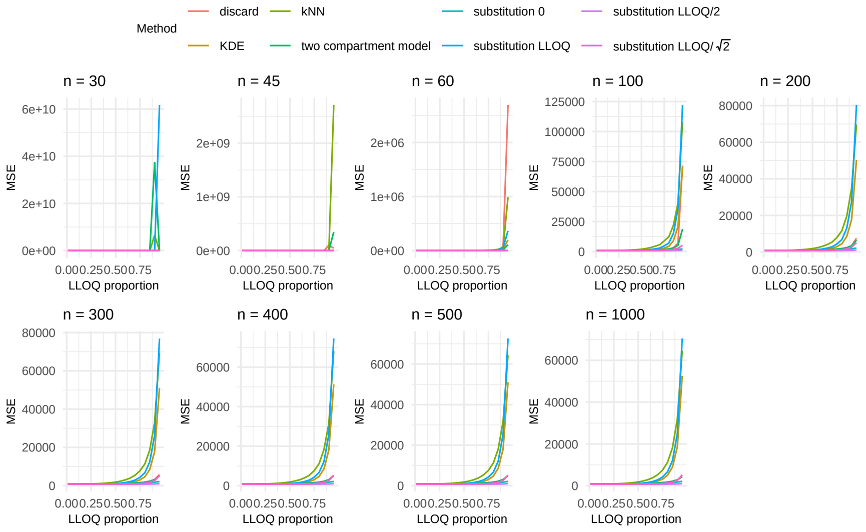

Figure S15: Results of the MSE for Model 1 with an $R^2 = 0.6$ for Setting S5. The colors refer to the different methods addressing BLOQ values. LLOQ = lower limit of quantification.

S2.1.2 Results for $R^2_{adj} = 0.1$

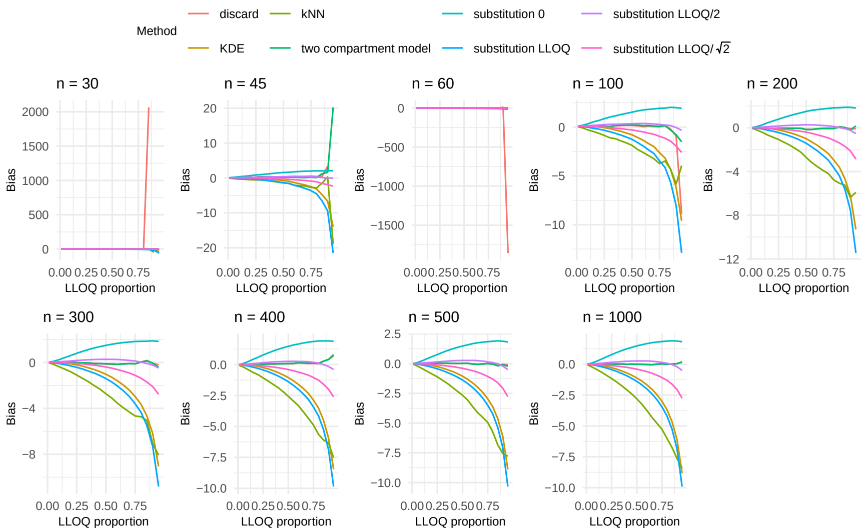

Figure S16: Results of the bias for Model 1 with an $R^2 = 0.1$ for Setting S1. The colors refer to the different methods addressing BLOQ values. LLOQ = lower limit of quantification.

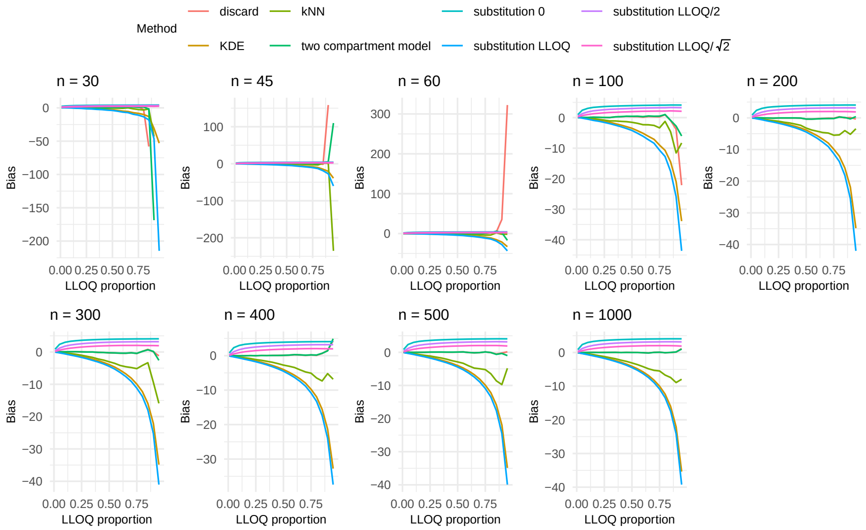

Figure S17: Results of the bias for Model 1 with an $R^2 = 0.1$ for Setting S2. The colors refer to the different methods addressing BLOQ values. LLOQ = lower limit of quantification.

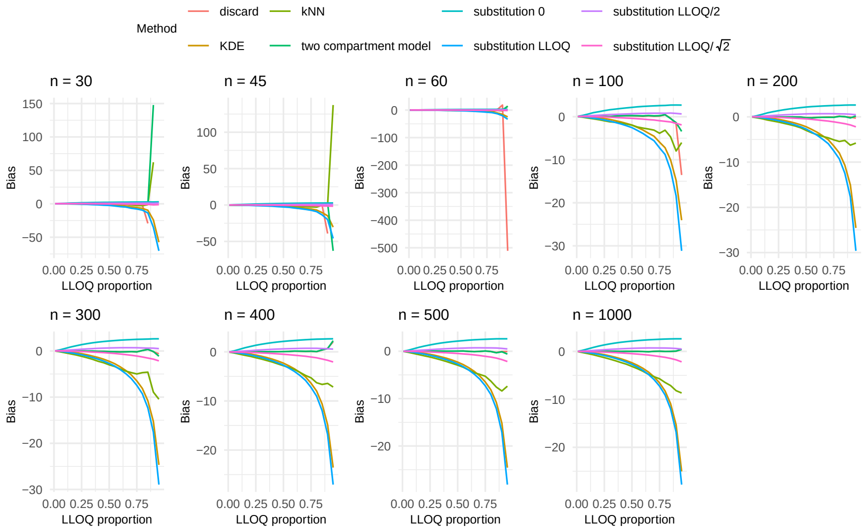

Figure S18: Results of the bias for Model 1 with an $R^2 = 0.1$ for Setting S3. The colors refer to the different methods addressing BLOQ values. LLOQ = lower limit of quantification.

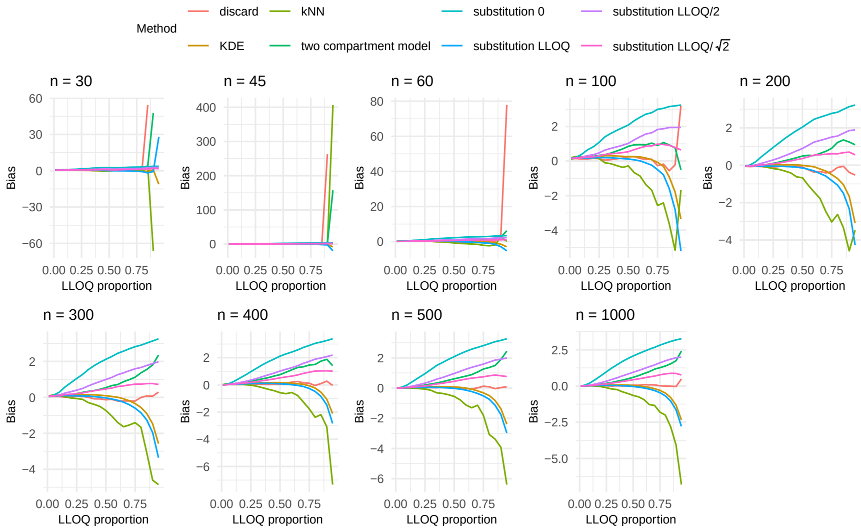

Figure S19: Results of the bias for Model 1 with an $R^2 = 0.1$ for Setting S4. The colors refer to the different methods addressing BLOQ values. LLOQ = lower limit of quantification.

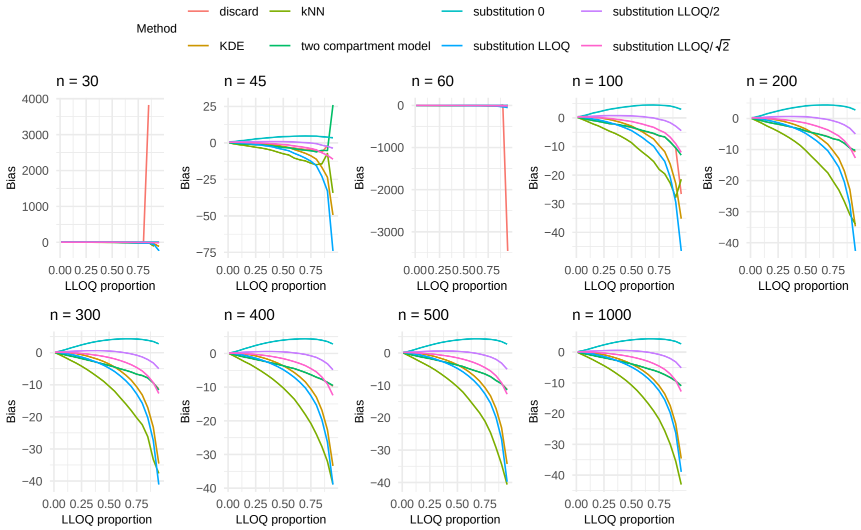

Figure S20: Results of the bias for Model 1 with an $R^2 = 0.1$ for Setting S5. The colors refer to the different methods addressing BLOQ values. LLOQ = lower limit of quantification.

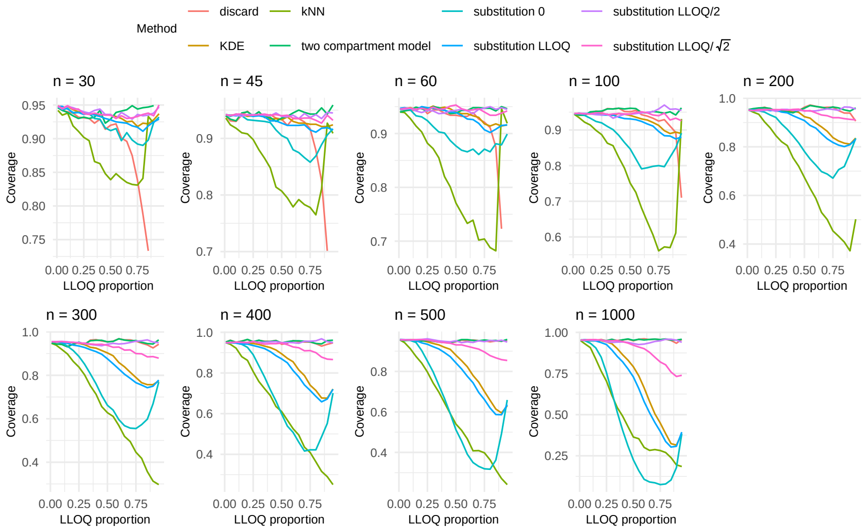

Figure S21: Results of the coverage for Model 1 with an $R^2 = 0.1$ for Setting S1. The colors refer to the different methods addressing BLOQ values. LLOQ = lower limit of quantification.

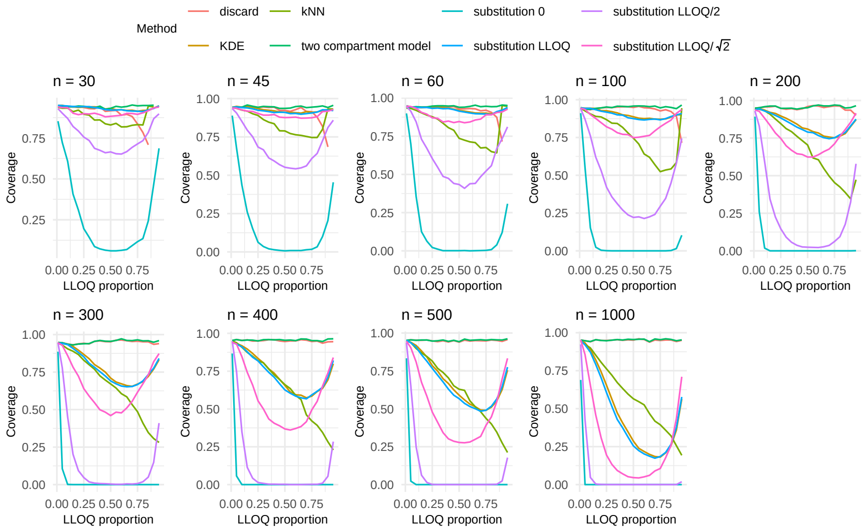

Figure S22: Results of the coverage for Model 1 with an $R^2 = 0.1$ for Setting S2. The colors refer to the different methods addressing BLOQ values. LLOQ = lower limit of quantification.

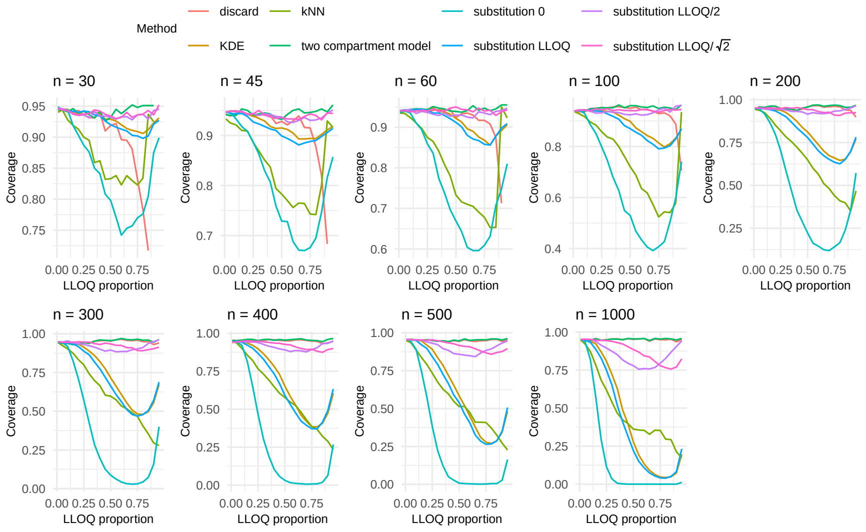

Figure S23: Results of the coverage for Model 1 with an $R^2 = 0.1$ for Setting S3. The colors refer to the different methods addressing BLOQ values. LLOQ = lower limit of quantification.

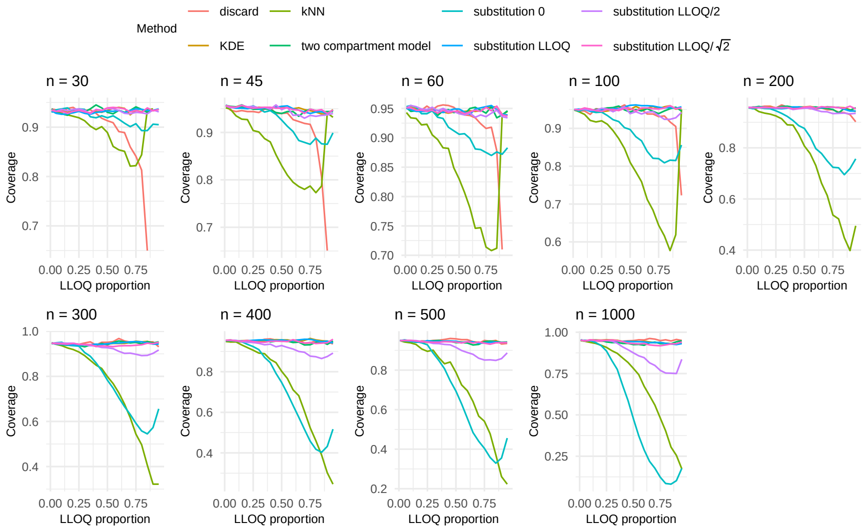

Figure S24: Results of the coverage for Model 1 with an $R^2 = 0.1$ for Setting S4. The colors refer to the different methods addressing BLOQ values. LLOQ = lower limit of quantification.

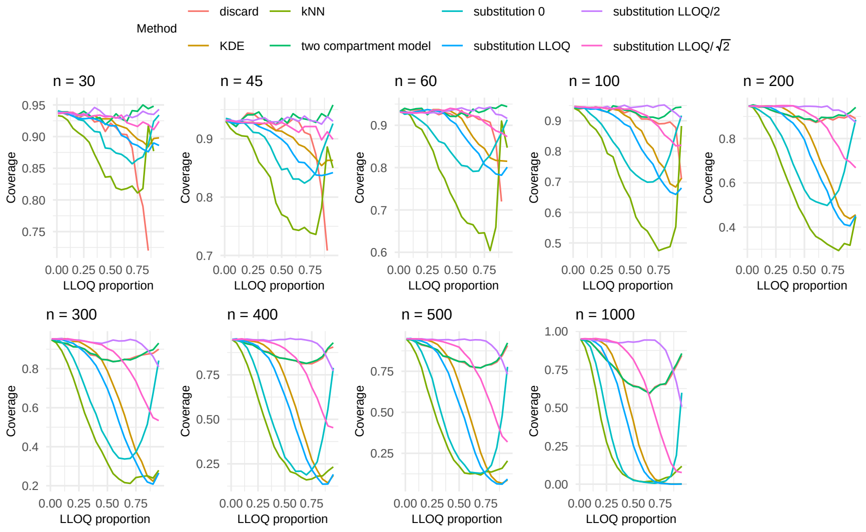

Figure S25: Results of the coverage for Model 1 with an $R^2 = 0.1$ for Setting S5. The colors refer to the different methods addressing BLOQ values. LLOQ = lower limit of quantification.

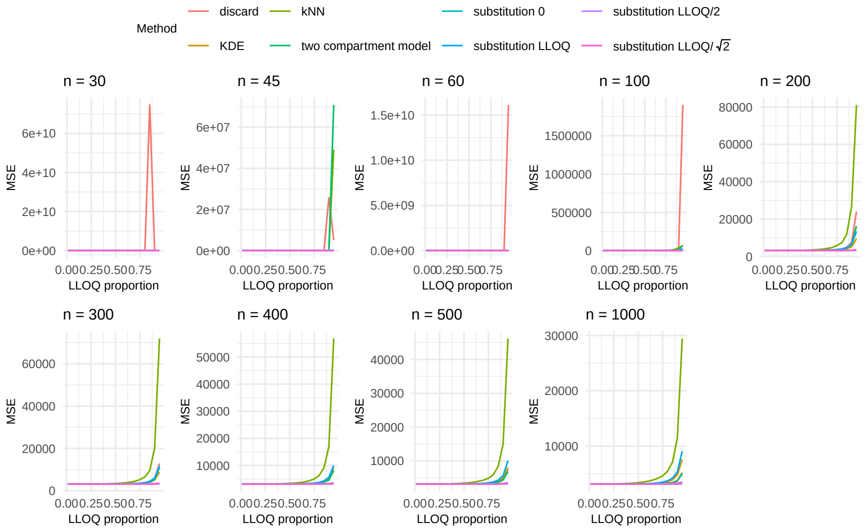

Figure S26: Results of the MSE for Model 1 with an $R^2 = 0.1$ for Setting S1. The colors refer to the different methods addressing BLOQ values. LLOQ = lower limit of quantification.

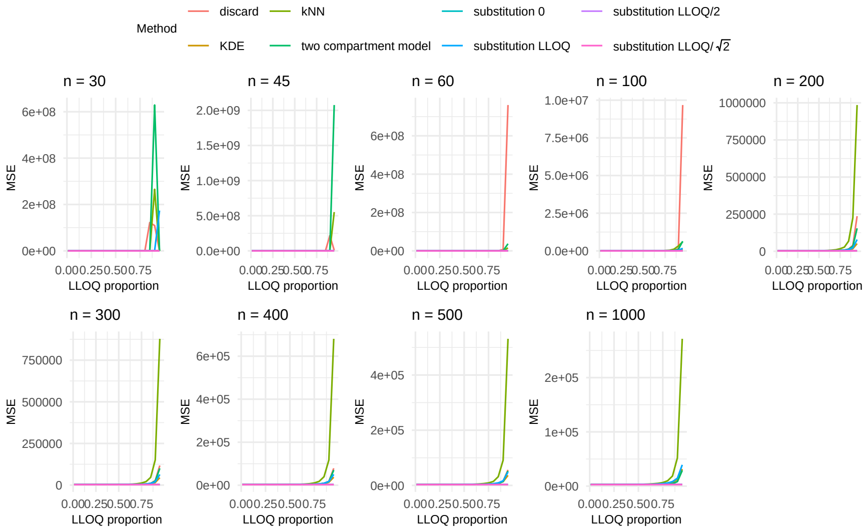

Figure S27: Results of the MSE for Model 1 with an $R^2 = 0.1$ for Setting S2. The colors refer to the different methods addressing BLOQ values. LLOQ = lower limit of quantification.

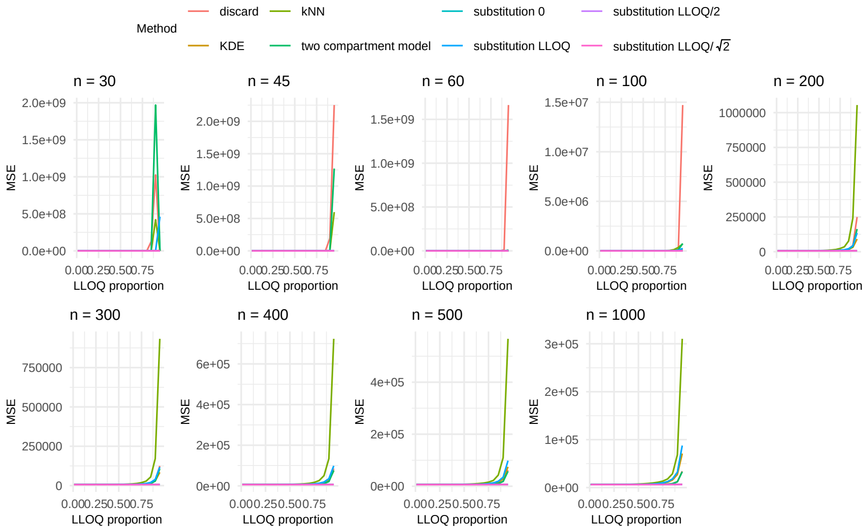

Figure S28: Results of the MSE for Model 1 with an $R^2 = 0.1$ for Setting S3. The colors refer to the different methods addressing BLOQ values. LLOQ = lower limit of quantification.

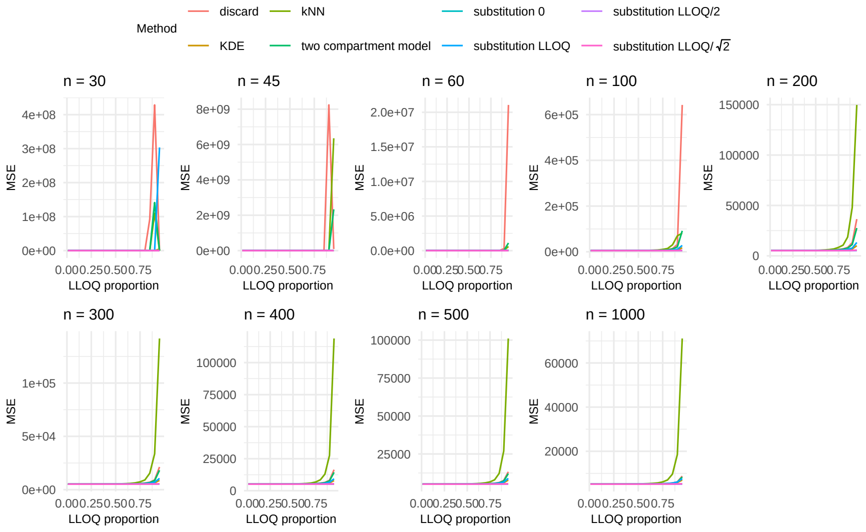

Figure S29: Results of the MSE for Model 1 with an $R^2 = 0.1$ for Setting S4. The colors refer to the different methods addressing BLOQ values. LLOQ = lower limit of quantification.

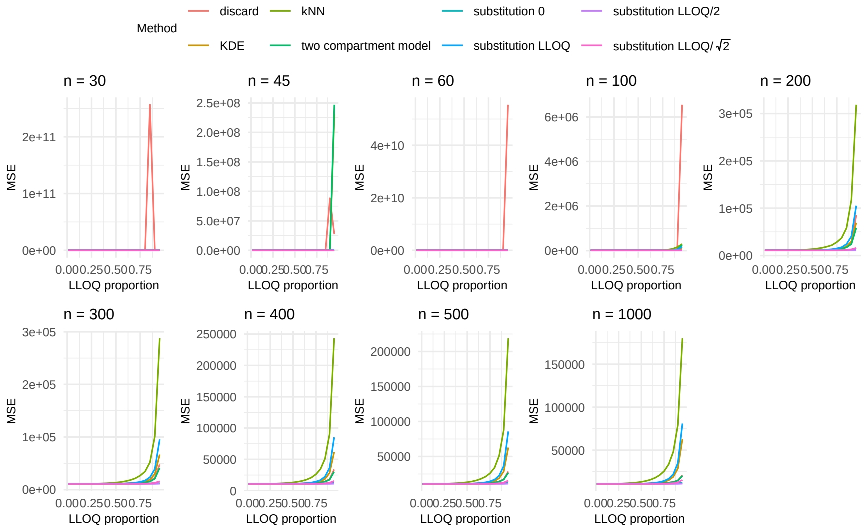

Figure S30: Results of the MSE for Model 1 with an $R^2 = 0.1$ for Setting S5. The colors refer to the different methods addressing BLOQ values. LLOQ = lower limit of quantification.

S2.2 Bias and coverage performance of Model 2: BLOQ variable as dependent variable

S2.2.1 Results for $R^2_{adj} = 0.6$

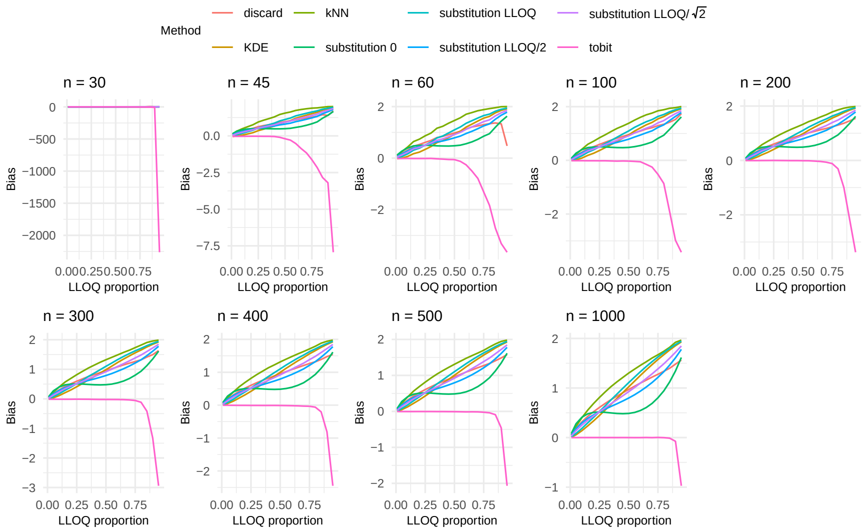

Figure S31: Results of the bias for Model 2 with an $R^2 = 0.6$ for Setting S1. The colors refer to the different methods addressing BLOQ values. LLOQ = lower limit of quantification.

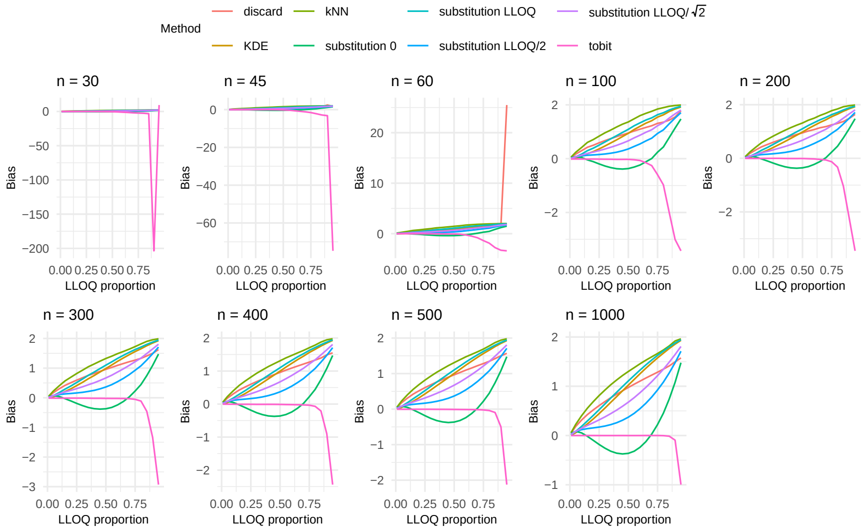

Figure S32: Results of the bias for Model 2 with an $R^2 = 0.6$ for Setting S2. The colors refer to the different methods addressing BLOQ values. LLOQ = lower limit of quantification.

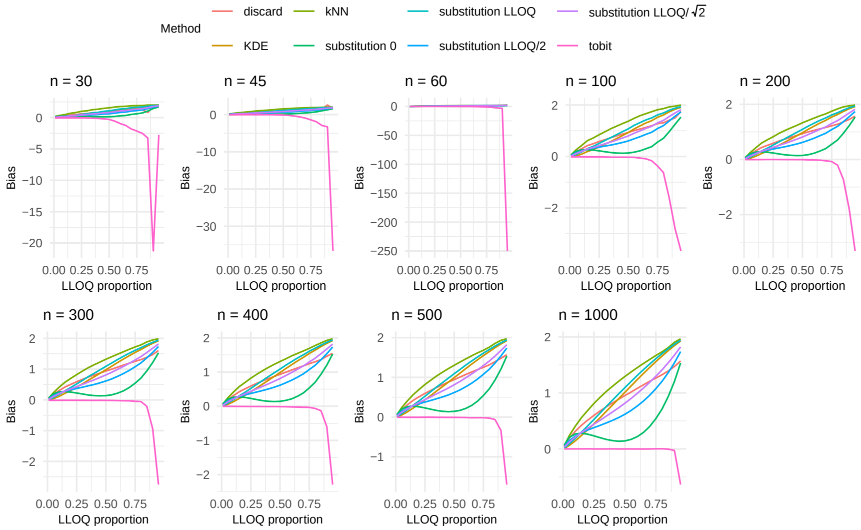

Figure S33: Results of the bias for Model 2 with an $R^2 = 0.6$ for Setting S3. The colors refer to the different methods addressing BLOQ values. LLOQ = lower limit of quantification.

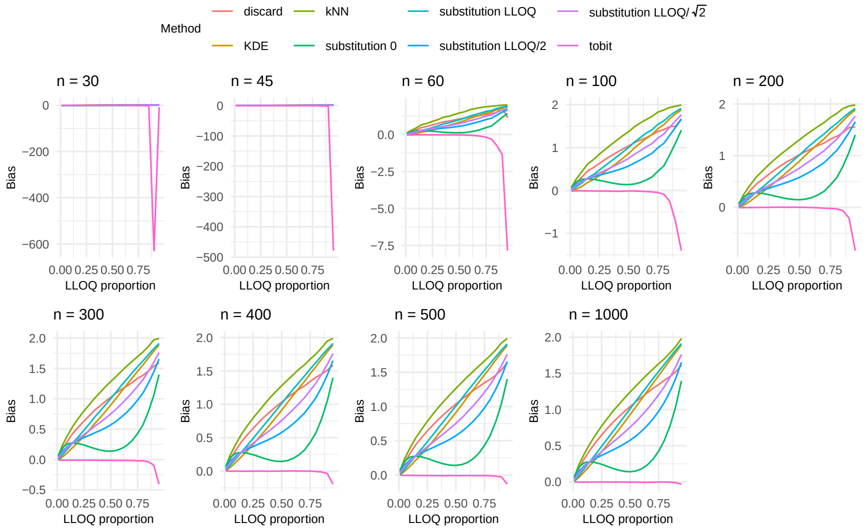

Figure S34: Results of the bias for Model 2 with an $R^2 = 0.6$ for Setting S4. The colors refer to the different methods addressing BLOQ values. LLOQ = lower limit of quantification.

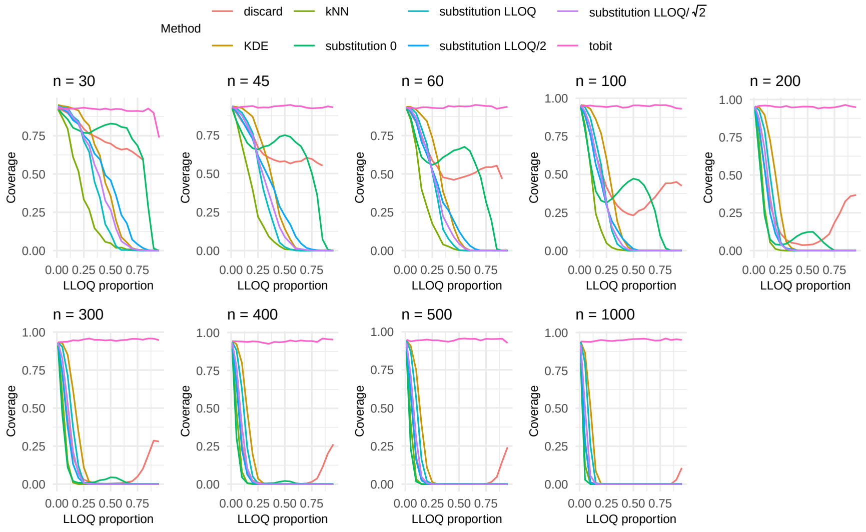

Figure S35: Results of the coverage for Model 2 with an $R^2 = 0.6$ for Setting S1. The colors refer to the different methods addressing BLOQ values. LLOQ = lower limit of quantification.

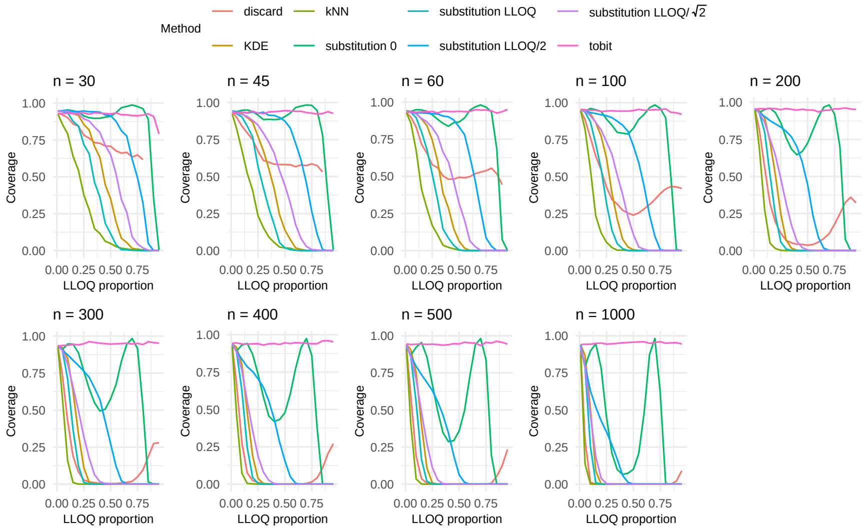

Figure S36: Results of the coverage for Model 2 with an $R^2 = 0.6$ for Setting S2. The colors refer to the different methods addressing BLOQ values. LLOQ = lower limit of quantification.

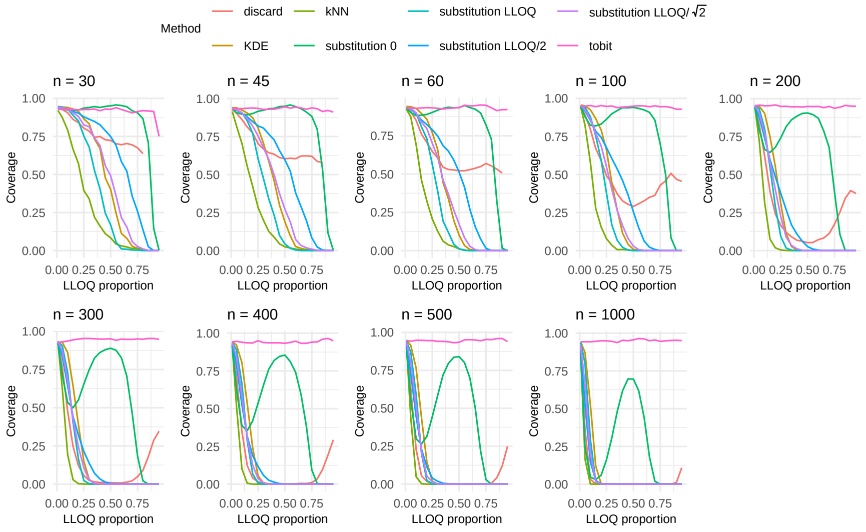

Figure S37: Results of the coverage for Model 2 with an $R^2 = 0.6$ for Setting S3. The colors refer to the different methods addressing BLOQ values. LLOQ = lower limit of quantification.

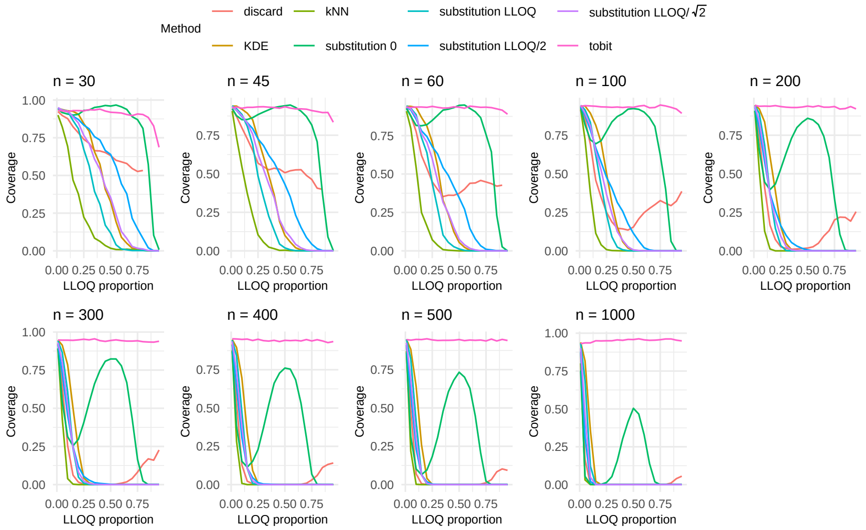

Figure S38: Results of the coverage for Model 2 with an $R^2 = 0.6$ for Setting S4. The colors refer to the different methods addressing BLOQ values. LLOQ = lower limit of quantification.

S2.2.2 Results for $R^2_{adj} = 0.1$

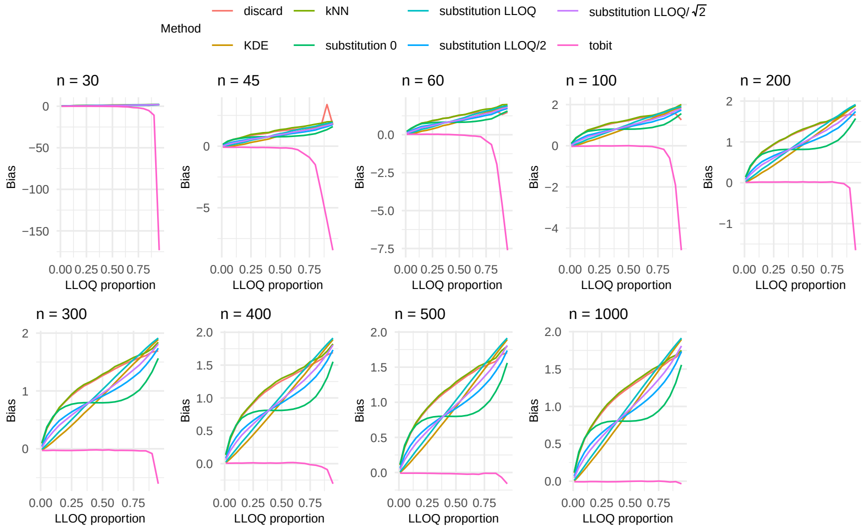

Figure S39: Results of the bias for Model 2 with an $R^2 = 0.1$ for Setting S1. The colors refer to the different methods addressing BLOQ values. LLOQ = lower limit of quantification.

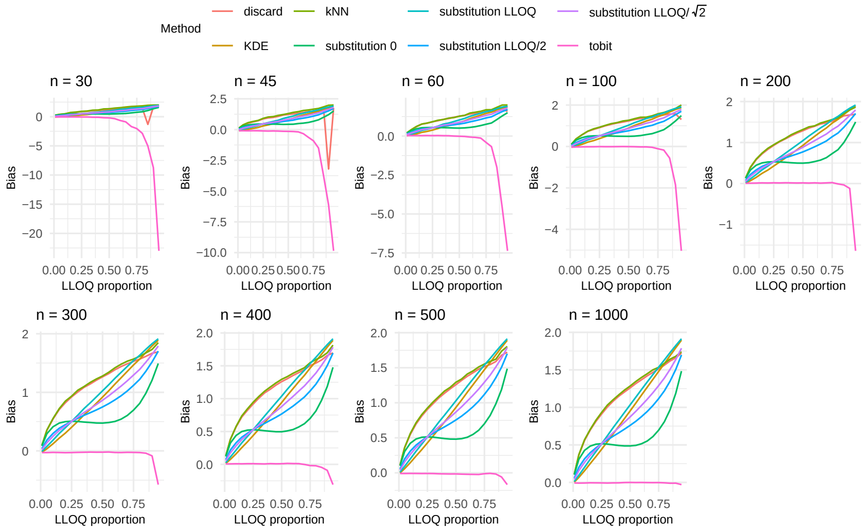

Figure S40: Results of the bias for Model 2 with an $R^2 = 0.1$ for Setting S2. The colors refer to the different methods addressing BLOQ values. LLOQ = lower limit of quantification.

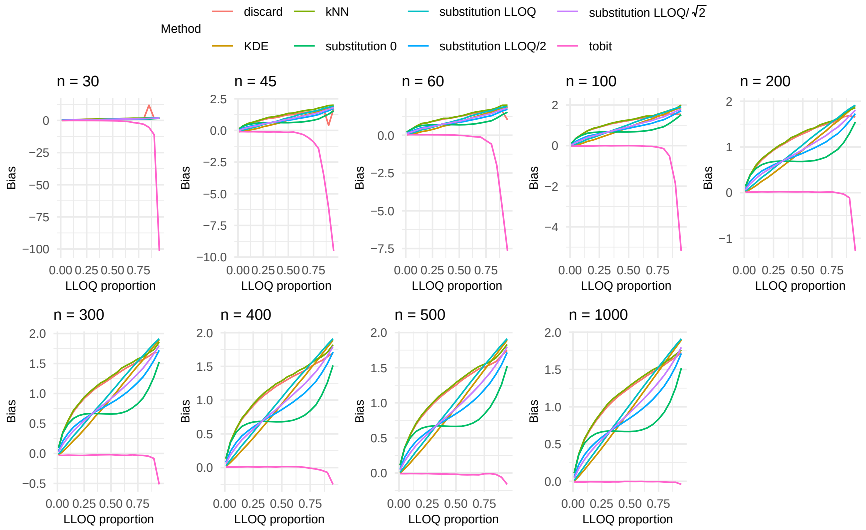

Figure S41: Results of the bias for Model 2 with an $R^2 = 0.1$ for Setting S3. The colors refer to the different methods addressing BLOQ values. LLOQ = lower limit of quantification.

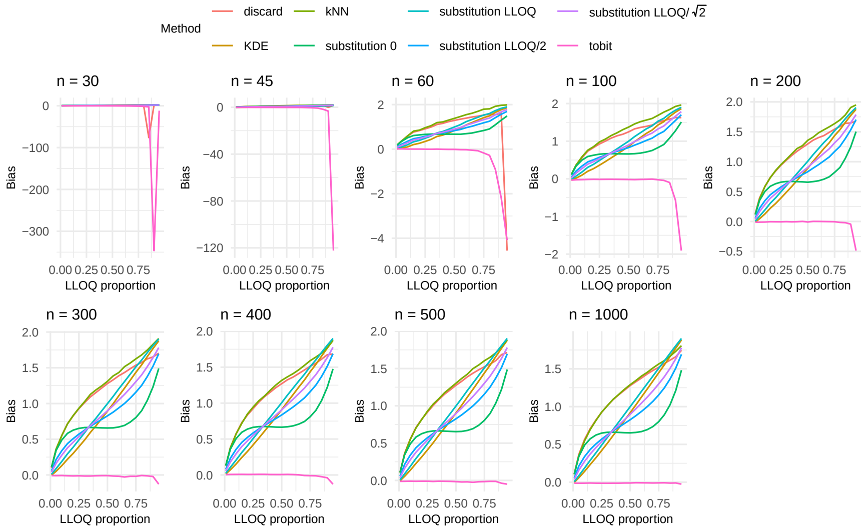

Figure S42: Results of the bias for Model 2 with an $R^2 = 0.1$ for Setting S4. The colors refer to the different methods addressing BLOQ values. LLOQ = lower limit of quantification.

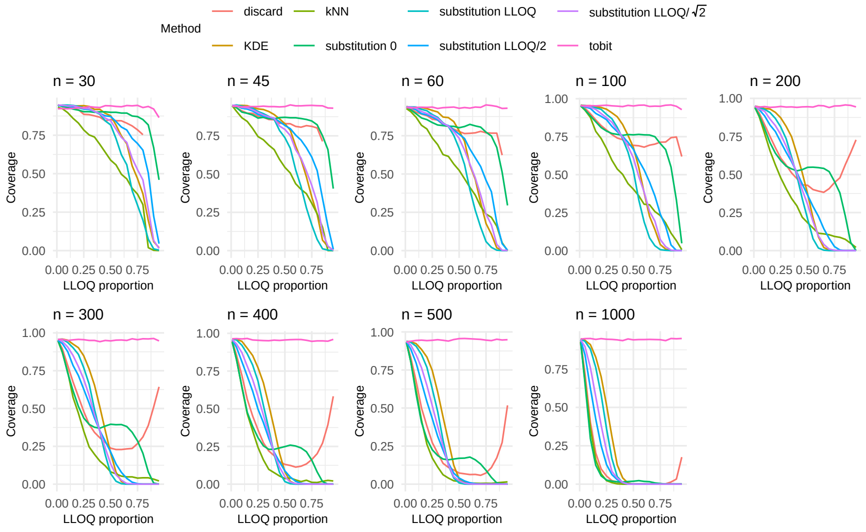

Figure S43: Results of the coverage for Model 2 with an $R^2 = 0.1$ for Setting S1. The colors refer to the different methods addressing BLOQ values. LLOQ = lower limit of quantification.

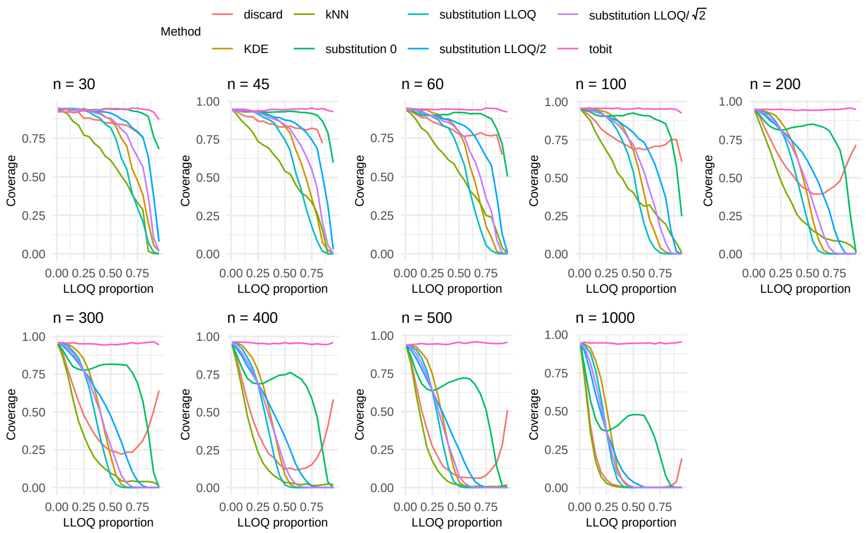

Figure S44: Results of the coverage for Model 2 with an $R^2 = 0.1$ for Setting S2. The colors refer to the different methods addressing BLOQ values. LLOQ = lower limit of quantification.

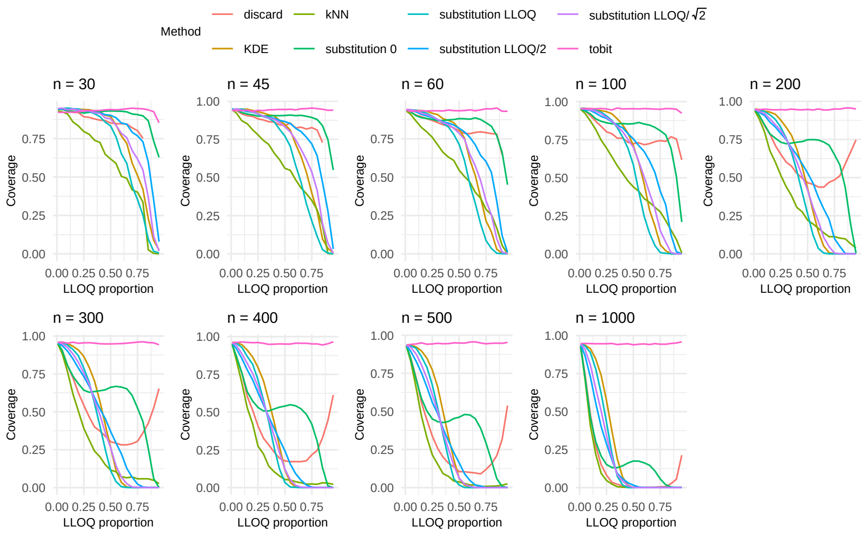

Figure S45: Results of the coverage for Model 2 with an $R^2 = 0.1$ for Setting S3. The colors refer to the different methods addressing BLOQ values. LLOQ = lower limit of quantification.

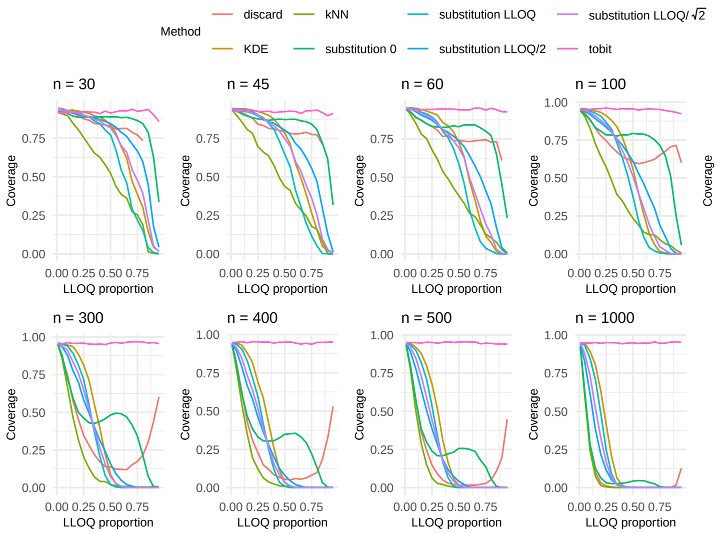

Figure S46: Results of the coverage for Model 2 with an $R^2 = 0.1$ for Setting S4. The colors refer to the different methods addressing BLOQ values. LLOQ = lower limit of quantification.
